# Transient Green’s Tensor for a Layered Solid Half-Space With Different Interface Conditions

**DOI:** 10.6028/jres.107.038

**Published:** 2002-10-01

**Authors:** Shu-Chu Ren, Nelson N. Hsu, Donald G. Eitzen

**Affiliations:** National Institute of Standards and Technology, Gaithersburg, MD 20899

**Keywords:** bond integrity, dynamic elasticity, fundamental solution, Green’s function, interface condition, layered half-space, NDE theoretical mechanics, wave mechanics

## Abstract

Pulsed ultrasonic techniques can be and have been used to examine the interface conditions of a bonded structure. To provide a theoretical basis for such testing techniques we model the structure as a layer on top of a half-space, both of different elastic properties, with various interface bonding conditions. The exact dynamic Green’s tensor for such a structure is explicitly derived from the three-dimensional equations of motion. The final solution is a series. Each term of the series corresponds to a successive arrival of a “generalized ray” and each is a definite line integral along a fixed path which can be easily computed numerically. Willis’ method is used in the derivation. A new scheme of automatic generation of the arrivals and ray paths using combinatorial analysis, along with the summation of the corresponding products of reflection coefficients is presented. A FORTRAN code is developed for computation of the Green’s tensor when both the source and the detector are located on the top surface. The Green’s tensor is then used to simulate displacements due to pulsed ultrasonic point sources of known time waveform. Results show that the interface bonding conditions have a great influence on the transient displacements. For example, when the interface bonding conditions vary, some of the first few head waves and regular reflected rays change polarities and amplitudes. This phenomenon can be used to infer the quality of the interface bond of materials in ultrasonic nondestructive evaluation. In addition the results are useful in the study of acoustic microscopy probes, coatings, and geo-exploration.

## 1. Introduction

The dynamic Green’s tensor of a structure is the fundamental solution to the transient mechanical wave problem of the structure. The theoretical prediction of the behavior of transient waves for arbitrary extended source distributions in space and time can be obtained once the time-domain dynamic Green’s tensor is known. The ability to predict the behavior of transient waves in a structure is important in the development and understanding of nondestructive evaluation techniques using ultrasonics or acoustic emission and in other problems of wave propagation in solid and liquid media.

In recent years, the “generalized ray” expansion technique has been applied to compute the Green’s tensor in a solid half-space or a solid infinite plate. The governing differential equation of motion is transferred to the Fourier or Laplace domain and the solution in the form of a series is obtained algebraically. Basically, there are two methods to invert the series from the transformed domain to the time-space domain. One is the well known Cagniard-de Hoop inversion method [1]; the other is a method developed by Willis in 1973 [2]. Willis’ method uses the Fourier transform and expresses the resulting transform as a series of “generalized rays” and then inverts the series term by term. For three-dimensional problems, as in the Cagniard-de Hoop method, only one integration remains. The integration path is always around a unit circle and is therefore “fixed” to some extent, but explicit evaluation of the integrand requires the numerical solution of an algebraic equation for each integration variable. Application of the Cagniard-de Hoop method requires a detailed discussion of the structure of a moderately complicated algebraic function accompanying the transform of the integration path. However, the integration path of the Willis inversion method is “fixed” and succeeds in avoiding the explicit discussion of the structure of the algebraic function and so is applied rather easily even in the anisotropic case. This is the main advantage of the Willis inversion method. The basis for carrying out the Willis inversion is that the solutions of elastodynamic problems are homogeneous functions of time, *t*, and position, *x*; that is, the problems are self-similar [2]. In addition, the displacements rather than the potentials are used, so the derivations are simplified, and the derivatives of the Green’s tensor about spatial coordinates are easily obtained as well. These derivatives can be interpreted as the displacements due to dipole sources. The three-dimensional transient Green’s tensor for an isotropic plate and the two-dimensional transient Green’s tensor for an anisotropic layer on an isotropic half-space were solved using Willis’ inversion method [3,4].

In this paper formulae are derived for computing the three-dimensional transient Green’s tensor for an isotropic layer overlay on an isotropic half-space. To understand the influence of the interface bonding condition on the behavior of transient waves, a welded interface, a liquid coupled interface, and a “vacuum” interface are considered. The results for the case of the liquid coupled interface are obtained by artificially casting the boundary conditions into a matrix form similar to that for the case of the welded interface. The results for the “vacuum” interface are obtained by considering the layer with no half-space. The last case has been computed and experimentally confirmed previously, thus it can be checked with independent results.

There are many rays which arrive at the observation point (detector) at the same time owing to the multiple reflection and the mode conversion of the incident *P* (longitudinal) ray or *S* (shear) ray emitted from the force source in the layer. These rays are kinematically equivalent and are called “kinematic analogs”. Obviously, it is not necessary to separately compute the contribution of each ray to the integration. The question of how many kinematically equivalent rays arrive at the detector at the same time for a given configuration is a problem of combinatorics. It is quite a complicated problem for a multiple layered solid half-space. So in this paper we present a new counting method to deal with this problem. In addition, some new numerical treatments are developed: 1) Automatic generation of the travel paths and the arrival times of various rays, 2) Automatic generation of the products of the reflection coefficients, and 3) An integration method for head wave rays.

The conditions for producing various head waves, surface waves, and interface waves are also examined. These conditions are determined from different singularities in the integrand.

A FORTRAN program has been developed for numerical computations of the response for any choice of materials for the layer and substrate. The computed results for the case of a plexiglass layer and glass substrate show that changes of the interface bonding condition have a great influence on the behavior of transient waves when both the source and detector are located on the top surface of the layer. For example, some of the first few head waves and regular reflected rays change their polarities and amplitudes when the interface bonding conditions change. This phenomenon can be used to infer the quality of the interface bonding of materials in ultrasonic nondestructive evaluation. In addition, results from this fundamental solution are expected to provide insight into the study and optimization of probing tools such as acoustic microscopes and in applications ranging from the study of coatings to geo-exploration.

## 2. Governing Equations and Boundary Conditions

**Fig. 1:**
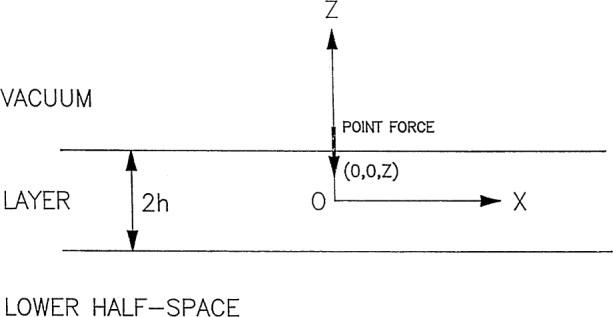
Schematic representation of a layered half-space.

Consider an elastic structure consisting of an anisotropic homogeneous layer of thickness 2*h* on a homogeneous half-space as shown in Fig. 1 and suppose there is a point force source of step function time dependence inside the layer. Then the fields in the layer satisfy the equations of motion

$$\begin{array}{c} \frac{\partial \sigma_{ij}^{I}}{\partial x_{j}}+f_{i}\delta (x_{1})\delta (x_{2})\delta (x_{3}-z)H(t)=\rho^{I}\frac{\partial^{2}{u^{I}}_{i}}{\partial t^{2}},\\ -h<x_{3}<h \end{array}$$ (1.1)

where (0, 0, *z*) are the position coordinates of the source. The origin of the Cartesian coordinates is at the center of the top layer; *u*^I^*_i_*, ***σ***^I^*_ij_*, and ***ρ***^I^ are the displacement components, the stress components, and the density of layer ***I***, respectively; *f_i_*, ***H***(*t*), and **δ**(*x*) are the components of the point force source, Heaviside step function, and Dirac delta function, respectively. The summation convention is used.

The stress and displacement gradient in the layer are related by the generalized Hooke’s law

$${\sigma_{ij}}^{I}=c_{ijk\ell }^{I}\frac{\partial \;u_{k}^{I}}{\partial \;x_{\ell }}$$ (1.2)

where *c*^I^*_ijkℓ_* are the elastic constants in the layer.

Similarly, the field equations and stress and displacement gradient relation in the lower half-space can be written by replacing “**I**” with “**II**”, thus

$$\begin{array}{cc} \frac{\partial \;\sigma_{ij}^{II}}{\partial \;x_{j}}=\rho^{\mathrm{II}}\frac{\partial^{2}u_{i}^{\mathrm{II}}}{\partial \;t^{2}}, & x_{3}<-h. \end{array}$$ (1.3)

where ***σ***^II^*_ij_*, *u*^II^*_i_*, and ***ρ***^II^ are the stress components, the displacement components, and the density in the half-space.

The stress and displacement gradient in the half-space are related by

$${\sigma_{ij}}^{\mathrm{II}}=c_{ijk\ell }^{\mathrm{II}}\frac{\partial \;u_{k}^{\mathrm{II}}}{\partial \;x_{\ell }},$$ (1.4)

where c^II^*_ijkℓ_* are the elastic constants in the half-space.

In addition, the solution should also satisfy the following conditions:

$$\begin{array}{cc} {u_{k}}^{I}={u_{k}}^{\mathrm{II}}=0, & t<0,\\ {\sigma_{ij}}^{I}={\sigma_{ij}}^{\mathrm{II}}=0, & t<0, \end{array}$$ (1.5)

and

$$\begin{array}{cc} {u_{k}}^{\mathrm{II}}=0, & x_{3} \end{array}\to -\infty .$$ (1.6)

In what follows we consider two cases of the interface bonding condition, whereas the top surface boundary conditions remain the same.

Case 1.
  Suppose the interface is “welded”, whereas the top surface condition is traction free; we have:
  $$\begin{array}{cc} {\sigma_{i3}}^{I}=0, & x_{3}=h, \end{array}$$ (1.7)
  $$\begin{array}{cc} {\sigma_{i3}}^{I}=\sigma_{i3}^{\mathrm{II}}, & x_{3}=-h, \end{array}$$ (1.8)
  $$\begin{array}{cc} {u_{i}}^{I}={u_{i}}^{\mathrm{II}}, & x_{3}=-h. \end{array}$$ (1.9)
Case 2.
  Suppose the interface is intimately “liquid” coupled, while the top surface condition is again traction free. We have:
  $$\begin{array}{cc} \sigma_{i3}=0, & x_{3}=h, \end{array}$$ (1.10)
  $$\begin{array}{cc} \sigma_{33}^{I}=\sigma_{33}^{\mathrm{II}}, & x_{3}=-h, \end{array}$$ (1.11)
  $$\begin{array}{cc} \sigma_{13}^{I}=\sigma_{23}^{I}=0, & x_{3}=-h, \end{array}$$ (1.12)
  $$\begin{array}{cc} \sigma_{13}^{\mathrm{II}}=\sigma_{23}^{\mathrm{II}}=0, & x_{3}=-h, \end{array}$$ (1.13)
  $$\begin{array}{cc} u_{3}^{I}=u_{3}^{\mathrm{II}}, & x_{3}=h. \end{array}$$ (1.14)

## 3. Solution Method

The outline of the solution procedure for the problem can be described as follows. First, introduce the Green’s tensor and take its Fourier transform in time and space; then, expand the transformed Green’s tensor according to the eigenvector of the Christoffel matrix, and decompose the fields into downgoing waves and upgoing waves; third, use boundary conditions to iteratively get the solution in the transform domain in a form of “generalized ray” series; finally, use the Willis inversion technique to get the solution.

Defining the “Heaviside Green’s tensor”, ***G****_ij_*, the displacements in the layer can be expressed as

$$u_{i}^{I}=G_{ij}f_{j}.$$ (1.15)

Substituting Eqs. (1.2) and (1.15) into Eq. (1.1) gives

$$c_{ijk\ell }^{I}\frac{\partial^{2}G_{kp}}{\partial x_{j}\partial x_{1}}+\delta_{ip}\delta (x_{1})\delta (x_{2})\delta (x_{3}-z)H(t)=\rho^{I}\frac{\partial^{2}G_{ip}}{dt^{2}},$$ (1.16)

where **δ***_ip_* is the Kronecker delta.

The Green’s tensor in the layer may also be expressed as

$$G=G^{\infty }+G^{I},$$ (1.17)

where ***G*** ^∞^is the infinite body Heaviside Green’s tensor and ***G***^I^ is the “image” tensor in the layer formed from the waves reflected on the boundaries *x*_3_ = ±*h*. All matrices are denoted in bold capitals and vectors in bold small letters.

Define matrices *K*^L^(***ω***, *ξ*) and *C*^L^(*ξ*) with components

$$K_{ik}^{L}(\omega ,x)=\rho^{L}\omega^{2}\delta_{ik}-c_{ijk\ell }^{L}\xi_{j}\xi_{1},$$ (1.18)

$$\begin{array}{c} C_{ik}^{L}(x)=c_{i3k\ell }^{L}\xi_{\ell },\\ L=I,\mathrm{II}, \end{array}$$ (1.19)

where the vector **ξ**= (ξ_1_, ξ_2_, ξ_3_).

In terms of Eqs. (1.15)–(1.19), we obtain the equations involving *G*^∞^ and *G*^I^ which satisfy

$$K^{I}\left(\frac{\partial }{\partial t},\nabla \right)G^{\infty }=I\delta (x_{1})\delta (x_{2})\delta (x_{3}-z)H(t),$$ (1.20)

and

$$K^{I}\left(\frac{\partial }{\partial t},\;\nabla \right)G^{I}=0,$$ (1.21)

where *I* is the identity matrix. The Green’s tensor, ***G***^II^, in the half-space satisfies

$$K^{\mathrm{II}}\left(\frac{\partial }{\partial t},\;\nabla \right)\;G^{\mathrm{II}}=0.$$ (1.22)

And the boundary conditions corresponding to cases 1 and 2 become:

Case 1.
  $$\begin{array}{cc} C^{I}(\nabla )(G^{\infty }+G^{I})=0, & x_{3}=h, \end{array}$$ (1.23)
  $$\begin{array}{cc} C^{I}(\nabla )(G^{\infty }+G^{I})=C^{\mathrm{II}}(\nabla )G^{\mathrm{II}}, & x_{3}=-h, \end{array}$$ (1.24)
  $$\begin{array}{cc} G^{\infty }+G^{I}=G^{\mathrm{II}}, & x_{3}=-h, \end{array}$$ (1.25)
  where ***C***^I^**(**∇) and ***C***^II^(∇) are the matrix operators with components
  $$C_{ik}^{L}(\nabla )=c_{i3k\ell }^{L}\frac{\partial }{\partial x_{\ell }},\;L=I,\mathrm{II}.$$ (1.26)
Case 2.
  $$C^{I}(\nabla )(G^{\infty }+G^{I})=0,\;x_{3}=h,$$ (1.27)
  $$I_{1}C^{I}(\nabla )(G^{\infty }+G^{I})=I_{1}C^{\mathrm{II}}(\nabla )G^{I}=0,\;x_{3}=-h,$$ (1.28)
  $$I_{2}C^{I}(\nabla )(G^{\infty }+G^{I})=I_{2}C^{\mathrm{II}}(\nabla )G^{I}=0,\;x_{3}=-h,$$ (1.29)
  $$I_{3}C^{I}(\nabla )(G^{\infty }+G^{I})=I_{3}C^{\mathrm{II}}(\nabla )G^{I},\;x_{3}=-h,$$ (1.30)
  $$I_{3}(G^{\infty }+G^{I})=I_{3}G^{\mathrm{II}},\;x_{3}=-h,$$ (1.31)
  where
  $$I_{1}=\left[\begin{array}{ccc} 1 & 0 & 0\\ 0 & 0 & 0\\ 0 & 0 & 0 \end{array}\right],$$ (1.32)
  $$I_{2}=\left[\begin{array}{ccc} 0 & 0 & 0\\ 0 & 1 & 0\\ 0 & 0 & 0 \end{array}\right],$$ (1.33)
  and
  $$I_{3}=\left[\begin{array}{ccc} 0 & 0 & 0\\ 0 & 0 & 0\\ 0 & 0 & 1 \end{array}\right],$$ (1.34)

## 4. Ray Expansion

We follow the method developed in Refs. [2,3,4], but provide only an outline here. Defining the Fourier transform of ***G*** ^∞^by

$${\hat{G}}^{\infty }(\omega ,\xi )=\int_{0}^{\infty }dt\int \int_{-\infty }^{\infty }\int dx_{1}dx_{2}dx_{3}G^{\infty }(t,x)\exp\left[i(\xi x-\omega t)\right],$$ (2.1)

from Eq. (1.20) we then get

$$K^{I}(\omega ,\xi ){\hat{G}}^{\infty }(\omega ,\xi )=\frac{-i}{\omega }I\exp(i\xi_{3}z),$$ (2.2)

where *ω* is taken to have negative imaginary parts while **ξ**= (ξ_1_, ξ_2_, ξ_3_) has real components. It is easily shown that ***Ĝ***^∞^ is an analytic function of *ω* in the lower half of the *ω*-plane, and its inverse transform as a function of time *t* is equal to zero when *t* < 0.

In order to express the inverse of the matrix ***K***^I^(*ω*, *ξ*) in terms of its eigenvectors, we consider the Christoffel equation

$$K^{I}(\omega ,\xi )u_{r}=\Lambda_{r}(\omega ,\xi )u_{r},$$ (2.3)

where *Λ*_r_(*ω*, *ξ*) and *u*_r_ are the eigenvalues and eigenvectors.

For given real *ω*, *ξ*_1_, and *ξ*_2_, the equation

$$\det\;K^{I}(\omega ,\xi )=0$$ (2.4)

has six roots *ξ*_3_ = *ξ*_3_^N^ (*ω*, *ξ*_α_), *N* = ±1, 2, 3, α = 1, 2, which may either be real or occur in complex conjugate pairs. By means of the concept of Riemann surfaces, the six roots may be considered to define a single-valued algebraic function *ξ*_3_(*ω*, *ξ*_α_) by Eq. (2.4), if *ω* is allowed to range over the six sheets of its Riemann surface [2]. It can be shown by analytic continuation that when *Im*(*ω*) < 0, the algebraic function *ξ*_3_ has positive imaginary parts on the three sheets of *N* = –1, –2, –3 and has negative imaginary parts on the other three sheets of *N* = +1, +2, +3.

Now let us consider the eigenvalues. Since Λ*_N_* =*ρ*^I^(*ω*^2^ – *ω*_N_^2^), where *ω_N_* is inverse to ξ_3_*^N^*(*ω*, *ξ*_α_), let *ξ*_3_*^N^*(*ω_N_*, *ξ*_α_) or det***K***^I^(*ω ^N^*, ξ) = 0, and we obtain the six roots ± *ω_N_*, *N* = 1, 2, 3 and therefore the three eigenvalues ***Λ****_N_*.

Normalizing the eigenvectors so that *u_m_ u*_n_^T^ = δ*_mn_*, we have

$$I=\sum u_{r}u_{r}^{T}=UU^{T}$$ (2.5)

where the matrix *U* consists of the three column vectors *u*_r_, while *U*^T^ is the transpose of *U*. As a result we have

$${\hat{G}}^{\infty }(\omega ,\xi )=-\frac{i}{\omega }\sum_{r=1}^{3}\Lambda_{r}^{-1}u_{r}u_{r}^{T}\exp(i\xi_{3}z).$$ (2.6)

Using symmetry with respect to the *x*_3_ axis, and taking the Fourier transform of ***G***^I^ from (*t*, *x*_1_, *x*_2_, *x*_3_) to (*ω*, ξ_1_, ξ_2_, *x*_3_) we obtain for the fields in the layer

$$\hat{G}(\omega ,\xi )=\int_{0}^{\infty }dt\int \int_{-\infty }^{\infty }dx_{1}dx_{2}G^{I}(t,x)\exp\left[i(\xi_{\alpha }x_{\alpha }-\omega t)\right],$$ (2.7)

where

$$\xi_{\alpha }x_{\alpha }=\xi_{1}x_{1}+\xi_{2}x_{2}.$$ (2.8)

In what follows we consider the cases of downgoing waves and upgoing waves in the layer respectively.

1. Downgoing waves.
  Consider the case *x*_3_ < *z*. Here *z* is the position of the point force source. Taking the inverse Fourier transform of Eq. (2.6) about ξ_3_ gives
  $$\begin{array}{c} {\bar{G}}^{\infty }(\omega ,\xi_{1},\xi_{2},x_{3})\\ =\frac{-i}{2\pi \omega }\int_{-\infty }^{\infty }d\xi_{3}\sum_{r=1}^{3}\Lambda_{r}^{-1}u_{r}u_{r}^{T}\exp[-i\xi_{3}(x_{3}-z)]. \end{array}$$ (2.9)
  When *x*_3_ < *z*, *x*_3_ − *z* is negative. Then the integral can be evaluated by closing the contour in the upper half of the ξ_3_-plane and by using Cauchy’s residue theorem. This gives
  $$\begin{array}{c} {\bar{G}}^{\infty }(\omega ,\xi_{1},\xi_{2},x_{3})\\ =\frac{1}{\omega }\sum_{M=1}^{3}\frac{u_{M}^{-}u_{M}^{-T}\exp[-i\xi_{3}^{M-}(x_{3}-z)]}{{\left(\frac{\partial \Lambda (\omega ,\xi )}{\partial \xi_{3}}\right)}_{\xi_{3}=\xi_{3}^{M-}}} \end{array}$$ (2.10)
  where *ξ*_3_*^M^*^−^ = *ξ*_3_*^M^*^−^(*ω*, ξ_1_, ξ_2_) above, which are located in the upper half of the ξ_3_-plane when *Im*(*ω*) < 0. The subscript or superscript “−” denotes downgoing waves.
2. Upgoing waves.
  In the case *x*_3_ > *z*, the contour of integral Eq. (2.9) can be closed in the lower half-plane. Similarly we obtain
  $${\bar{G}}_{+}^{\infty }(\omega ,\xi_{1},\xi_{2},x_{3})=\frac{1}{\omega }\sum_{N=1}^{3}\frac{u_{N}^{+}u_{N}^{+T}\exp[-i\xi_{3}^{N+}(x_{3}-z)]}{{\left(\frac{\partial \Lambda (\omega ,\xi )}{\partial \xi_{3}}\right)}_{\xi_{3}=\xi_{3}^{N+}}},$$ (2.11)
  where *ξ_3_^N^*^+^ = *ξ_3_^N^*^+^(*ω*, *ξ*_1_, *ξ*_2_) are the three roots of Eq. (2.4) which are located in the lower half of the *ξ*_3_-plane, when *Im*(*ω*) < 0. The subscript or superscript “+” denotes upgoing waves.

The general solution of ***Ḡ***^∞^ (*ω*, *ξ*_1_, *ξ*_2_, *x*_3_) should include three downgoing plane waves and three upgoing plane waves. The reflected waves in the layer should also include the downgoing waves and the upgoing waves, but there exist only the downgoing waves in the lower half-space. Then we respectively obtain

$${\bar{G}}^{\infty }(\omega ,\xi_{1},\xi_{2},x_{3})={\bar{G}}_{-}^{\infty }(\omega ,\xi_{1},\xi_{2},x_{3})+{\bar{G}}_{+}^{\infty }(\omega ,\xi_{1},\xi_{2},x_{3}),$$ (2.12)

$$\begin{array}{c} {\bar{G}}^{I}=\sum_{M=1}^{3}u_{M}^{-}v_{M}^{-T}\exp[-i\xi_{3}^{M-}(x_{3}-h)]\\ +\sum_{N=1}^{3}u_{N}^{+}v_{N}^{+}\;\exp[-i\xi_{3}^{N+}(x_{3}-h)],\;-h<x_{3}<h, \end{array}$$ (2.13)

$${\bar{G}}^{II}=\sum_{P=1}^{3}q_{P}^{-}w_{P}^{-T}\exp[-i\xi_{3}^{P-}(x_{3}-h)],\;x_{3}<-h,$$ (2.14)

where $q_{P}^{-}$ represents the eigenvectors of an infinite body in the lower half-space while ${\xi^{P-}}_{3}$ represent the roots of Eq. (2.4) when the superscript “I” is replaced by “II”, for the same reasoning as mentioned above, which roots locate on the upper half-plane of *ξ*_3_-plane when *Im*(*ω*) < 0, and *v*^−^*_M_*, *v*^+^*_N_*, and *w*^−^*_P_* are the coefficients to be determined.

Further matrix notation is introduced as follows,

$$U^{-}=[u_{1}^{-},u_{2}^{-},u_{3}^{-}],$$ (2.15)

$$V^{-}=[v_{1}^{-},v_{2}^{-},v_{3}^{-}],$$ (2.16)

with corresponding definitions when superscript “−” is replaced by “+” and

$$Q^{-}=[q_{1}^{-},q_{2}^{-},q_{3}^{-}],$$ (2.17)

$$W^{-}=[w_{1}^{-},w_{2}^{-},w_{3}^{-}],$$ (2.18)

Then the previous equations can be written in more compact form as

$$\begin{array}{l} {\bar{G}}^{\infty }(\omega ,\xi_{1},\xi_{2},x_{3})=H(z-x_{3})U^{-}P^{-}(z-x_{3}-h)D^{-}U^{-T}\\ +H(x_{3}-z)U^{+}P^{+}(z-x_{3}+h)D^{+}U^{+T}, \end{array}$$ (2.19)

where *P*^−^, *P*^+^, *D*^−^, and *D*^+^ are diagonal matrices with components

$${\left(P^{-}\right)}_{k}^{\ell }(x_{3})=\delta_{k}^{\ell }\exp[i\xi_{3}^{\ell -}(x_{3}+h)],$$ (2.20)

where $\delta_{k}^{\ell }$ is the Kronicker delta. Also,

$${\left(P^{+}\right)}_{k}^{\ell }(x_{3})=\delta_{k}^{\ell }\exp[i\xi_{3}^{\ell +}(x_{3}-h)],$$ (2.21)

$${{\left(D^{-}\right)}_{k}^{\ell }=\delta_{k}^{\ell }{[\omega \left(\frac{\partial \Lambda (\omega ,\xi )}{\partial \xi_{3}}\right)}_{\xi_{3}=\xi_{3}^{\ell -}})}^{-1},$$ (2.22)

$${{\left(D^{+}\right)}_{k}^{\ell }=\delta_{k}^{\ell }{[\omega \left(\frac{\partial \Lambda (\omega ,\xi )}{\partial \xi_{3}}\right)}_{\xi_{3}=\xi_{3}^{\ell +}})}^{-1},$$ (2.23)

and

$${\bar{G}}^{I}=U^{-}P^{-}(-x_{3})V^{-T}+U^{+}P^{+}(-x_{3})V^{+T},$$ (2.24)

$${\bar{G}}^{\mathrm{II}}=Q^{-}S^{-}(-x_{3})W^{-T},$$ (2.25)

where *S*^−^ is the diagonal matrix with components

$${\left(S^{-}\right)}_{k}^{\ell }(x_{3})=\delta_{k}^{\ell }\exp[i\xi_{3}^{\ell -}(x_{3}-h)].$$ (2.26)

Case 1.
  If we are only interested in the fields in the layer, substituting Eqs. (2.19), (2.24), and (2.25) into the Fourier transformed equations corresponding to the boundary conditions Eqs. (1.23)–(1.25) and eliminating the coefficient matrix *W*^−^, we get
  $$B_{1}^{-}V^{-T}-B_{1}^{+}P^{+}V^{+T}=-B_{1}^{+}P^{+}(z)D^{+}U^{+T}$$ (2.27)
  and
  $$B_{2}^{+}V^{+T}-B_{2}^{-}P^{-}V^{-T}=-B_{2}^{-}P^{-}(z)D^{-}U^{-T}$$ (2.28)
  where
  $$B_{1}^{+}=C^{I}(\xi )U^{+},$$ (2.29)
  $$B_{2}^{+}=[C^{I}(\xi )-C^{\mathrm{II}}(\xi )Q^{-}{\left(Q^{-}\right)}^{-1}]U^{+},$$ (2.30)
  $$B_{1}^{-}=C^{I}(\xi )U^{-},$$ (2.31)
  $$B_{2}^{-}=[C^{I}(\xi )-C^{\mathrm{II}}(\xi )Q^{-}{\left(Q^{-}\right)}^{-1}]U^{-},$$ (2.32)
  $$P^{+}=-P^{+}(-h),$$ (2.33)
  $$P^{-}=-P^{-}(h).$$ (2.34)
  As *h* approaches infinity the exponential functions *P*^+^ and *P*^−^ on the left-hand sides of Eqs. (2.27) and (2.28) approach zero because the exponential factors have negative and positive imaginary parts, respectively. Therefore, Eqs. (2.27) and (2.28) uncouple into two independent equations and the problem becomes two separate problems for half-spaces *x*_3_ < *h* and *x*_3_ > −*h*. In addition, the larger *Im*(*ω*) becomes the smaller exponential functions *P*^+^ and *P*^−^, and so the equations can be solved iteratively to give a uniformly convergent series of “generalized rays” for given *ξ*_1_, *ξ*_2_, and the real part of *ω*. Finding *V*^−T^ and *V*^+T^ from Eqs. (2.27) and (2.28) by means of an iteration technique similar to Ref. [5] and substituting the results obtained into the previous relevant equations we get the general field in the layer
  $$\bar{G}={\bar{G}}^{-}+{\bar{G}}^{+},$$ (2.35)
  where
  $$\begin{array}{c} {\bar{G}}^{-}=H(z-x_{3})U^{-}P^{-}(z-x_{3}-h)S^{-}\\ -U^{-}P^{-}(-x_{3})R^{+}P^{+}(z)S^{+}-U^{-}P^{-}(-x_{3})R^{+}P^{+}R^{-}P^{-}(z)S^{-}\\ -U^{-}P^{-}(-x_{3})R^{+}P^{+}R^{-}P^{-}R^{+}P^{+}(z)S^{+}-\ldots \end{array}$$ (2.36)
  and
  $$\begin{array}{c} {\bar{G}}^{+}=H(x_{3}-z)U^{+}P^{+}(z-x_{3}+h)S^{+}\\ -U^{+}P^{+}(-x_{3})R^{-}P^{-}(z)S^{-}-U^{+}P^{+}(-x_{3})R^{-}P^{-}R^{+}P^{+}(z)S^{+}\\ -U^{+}P^{+}(-x_{3})R^{-}P^{-}R^{+}P^{+}R^{-}P^{-}(z)S^{-}-\ldots \end{array}$$ (2.37)
  which are the downgoing waves and the upgoing waves in the layer, respectively. Their physical meanings are obvious. If we notice that *R*^+^ and *R*^−^ are respectively the matrices of the reflection coefficients at the top surface and the lower surface of the layer, and the diagonal matrices *P*^+^ and *P*^−^ represent phase delays, then the first term of Eq. (2.36) represents the downgoing direct rays from the source to the observation point (detector), the second term describes the rays which reflect once at the top surface of the layer and so on. Similarly, we can explain each term of Eq. (2.37). Using the concept of an infinite linear array of image sources, then, with the exception of the first (direct wave) term, all the other terms of Eqs. (2.36) or (2.37) can be considered to be produced from the corresponding image sources located above or below the layer. The definitions of the notation of Eqs. (2.36) and (2.37) are as follows:
  $$L_{1}^{-}={\left(B_{1}^{-}\right)}^{-1},\;L_{2}^{+}={\left(B_{2}^{+}\right)}^{-1},$$ (2.38)
  $$R^{-}=L_{2}^{+}B_{2}^{-},\;R^{+}=L_{1}^{-}B_{1}^{+},$$ (2.39)
  $$S^{-}=D^{-}U^{-T},\;S^{+}=D^{+}U^{+T}.$$ (2.40)
  A typical term of Eq. (2.36) except for the first term may be written as
  $$U^{-}P^{-}(-x_{3})R^{+}P^{+}R^{-}P^{-}\ldots R^{(-1)k}P^{(-1)k}(z)S^{(-1)k},$$ (2.41)
  where *k* counts the number of *P*’s, *R*’s or *S*’s in the term. Interchanging superscripts “+” and “−” in Eq. (2.41) gives the typical term of Eq. (2.37).
  Suppose an element of the matrix of a typical term of Eq. (2.41) is written in the form
  $$F(\omega ,\xi )\exp[-i(\xi_{\alpha }x_{\alpha }-\omega t+\lambda_{1}\xi_{3}^{M}+\lambda_{2}\xi_{3}^{N})].$$
  Then the inverse Fourier transform of Eq. (2.36) gives
  $$\begin{array}{l} {\left(G^{-}\right)}^{ml}\frac{1}{8\pi^{3}}\int_{-\infty -0i}^{\infty -0i}d\omega \int \int d\xi_{1}d\xi_{2}\sum F(\omega ,\xi )\\ \exp[-i(\xi_{\alpha }x_{\alpha }-\omega t+\lambda_{1}\xi_{3}^{M}+\lambda_{2}\xi_{3}^{N})], \end{array}$$ (2.42)
  where (*G*^−^)*^ml^* is the element of the matrix *G*^−^ and *F*(*ω*, *ξ*) is a homogeneous function of degree −2, because the elements of the matrices *U*^±^ and *R*^±^ are homogeneous functions of degree zero, while the elements of the matrices *S*^±^ are homogeneous functions of degree −2. In addition, since the series of “generalized rays” is uniformly convergent, we can invert the integration term by term using the Willis inversion method (Appendix A) to obtain
  $${\left(G^{-}\right)}^{m\ell }=\frac{-1}{4\pi^{2}}\sum \oint_{\begin{array}{l} \left|h\right|=1\\ \phi =0 \end{array}}\text{ds}\frac{F(\Omega ,\eta_{\alpha })}{-t+\lambda_{1}\xi_{3,\Omega }^{M}+\lambda_{2}\xi_{3,\Omega }^{N}},$$ (2.43)
  where (*G*^−^)*^mℓ^* is the element of *G*^−^, while the subscript *Ω* denotes ∂/∂*Ω* and
  $$\Omega =\frac{\omega }{\left|\xi \right|},\;\eta_{\alpha }=\frac{\xi_{\alpha }}{\left|\xi \right|}.$$ (2.44)
  *Ω* in the integrand is now taken as the root in the lower half-plane of the equation
  $$-\Omega t+\eta_{\alpha }x_{\alpha }+\lambda_{1}\xi_{3}^{M}(\Omega ,\eta_{\alpha })+\lambda_{2}\xi_{3}^{N}(\Omega ,\eta_{\alpha })=\varepsilon i,$$ (2.45)
  where *ε* is an arbitrary infinitesimal number.
  For the elements of *G*^+^, we have similar results.
Case 2.
  In this case, substituting Eqs. (2.19), (2.24), and (2.25) into the Fourier transformed equations corresponding to the boundary conditions Eqs. (1.27)–(1.29), we get the same Eqs. (2.27) and (2.28) when *B*^+^_2_ and *B*^−^_2_ are replaced by B*^+^_2_ and B*^−^_2_, respectively, and
  $$\begin{array}{c} B^{*\pm }=\{C^{I}(\xi )-(I_{3}C^{\mathrm{II}}(\xi )Q^{-})\\ {\left[(I_{1}+I_{2})C^{\mathrm{II}}(\xi )Q^{-}+I_{3}Q^{-}\right]}^{-1}\}U^{\pm }. \end{array}$$ (2.46)
  Defining
  $$L_{2}^{*+}={\left(B_{2}^{*+}\right)}^{-1},$$ (2.47)
  $$R^{*-}=L_{2}^{*+}B_{2}^{*-},$$ (2.48)
  and using the matrices with “*” instead of the corresponding matrices without “*” in Eqs. (2.35)–(2.36), we immediately obtain the solution of case 2. The inversion of the transform is the same.

Up to now we have not considered the properties of the materials, so all the previous results can be applied to the general case of arbitrary anisotropic materials.

## 5. Special Case of Isotropic Materials

When the layer and the lower half-space consist of two different isotropic materials, then the components of *K*^L^(*ω*, *ξ*) become

$$\begin{array}{c} K_{ik}^{L}(\omega ,\xi )\\ =(\rho_{L}\omega^{2}-\mu_{L}\xi_{j}\xi_{j})\delta_{ik}-(\lambda_{L}+\mu_{L})\xi_{i}\xi_{k},\;L=I,\;\mathrm{II}. \end{array}$$ (3.1)

where *λ*_L_ and *μ*_L_ are Lame’s elastic constants of the materials in the layer (L = I) and the lower half-space (L = II).

The roots of the equations

$$\det\;K^{L}(\omega ,\xi )=0,\;L=I,\;\mathrm{II}$$ (3.2)

are

$$\xi_{3}^{\pm aL}=\pm p_{L}\;(\text{once}),$$ (3.3)

$$\xi_{3}^{\pm cL}=\pm q_{L}\;(\text{twice}),$$ (3.4)

where

$$p_{L}=i{\left(\xi^{2}-\frac{\omega^{2}}{a_{L}^{2}}\right)}^{1/2},\;L=I,\mathrm{II},$$ (3.5)

$$q_{L}=i{\left(\xi^{2}-\frac{\omega^{2}}{c_{L}^{2}}\right)}^{1/2},\;L=I,\mathrm{II},$$ (3.6)

$$a_{L}^{2}=\left(\lambda_{L}+\mu_{L}\right)/\rho_{L},\;L=I,\;\mathrm{II},$$ (3.7)

$$c_{L}^{2}=\mu_{L}/\rho_{L},\;L=I,\mathrm{II},$$ (3.8)

$$\xi^{2}=\xi_{1}^{2}+\xi_{2}^{2}.$$ (3.9)

Making a comparison with the previous sections gives

$$\begin{array}{c} \xi_{3}^{M-}=p_{I},\;q_{I},\;q_{I}\\ \xi_{3}^{N+}=-p_{I},-q_{I},-q_{I}. \end{array}$$ (3.10)

Three roots, *p*_I_, *q*_I_, and *q*_I_, of the six roots in Eq. (3.10) are in the upper half of the *ξ*-plane, whereas the other three roots, −*p*_I_, −*q*_I_, and −*q*_I_, are in the lower half of the *ξ*_3_-plane. For the details of the distribution of these roots, refer to Ref. [2]

The normalized eigenvectors corresponding to the roots of Eq. (3.10) are

$$u_{1}^{\pm }=\left[\begin{array}{c} \xi_{1}\\ \xi_{2}\\ \pm p_{I} \end{array}\right]{\left(\xi^{2}+{\left(p_{I}\right)}^{2}\right)}^{-1/2},$$ (3.11)

$$u_{2}^{\pm }=\left[\begin{array}{c} \mp \xi_{1}q_{I}\\ \mp \xi_{2}q_{I}\\ -\xi^{2} \end{array}\right]{\left(\xi^{2}+{\left(q_{I}\right)}^{2}\right)}^{-1/2}\xi^{-1},$$ (3.12)

and

$$u_{3}^{\pm }=\left[\begin{array}{c} -\xi_{2}\xi^{-1}\\ \xi_{1}\xi^{-1}\\ 0 \end{array}\right].$$ (3.13)

For the lower half-space there exist only the downgoing waves; the following three roots should be chosen as

$$\xi_{3}^{p-}=p_{\mathrm{II}},q_{\mathrm{II}},q_{\mathrm{II}}.$$ (3.14)

The corresponding eigenvectors are

$$q_{1}^{-}=\left[\begin{array}{c} \xi_{1}\\ \xi_{2}\\ p_{\mathrm{II}} \end{array}\right]{\left[\xi^{2}+{\left(p_{\mathrm{II}}\right)}^{2}\right]}^{-1/2},$$ (3.15)

$$q_{2}^{-}=\left[\begin{array}{c} \xi_{1}q_{\mathrm{II}}\\ \xi_{2}q_{\mathrm{II}}\\ -\xi^{2} \end{array}\right]{\left[\xi^{2}+{\left(q_{\mathrm{II}}\right)}^{2}\right]}^{-1/2}/\xi ,$$ (3.16)

and

$$q_{3}^{-}=\left[\begin{array}{c} -\xi_{2}\xi^{-1}\\ \xi_{1}\xi^{-1}\\ 0 \end{array}\right].$$ (3.17)

The matrix operators *C*^I^(*ξ*) and *C*^II^(*ξ*) should always operate on the respective regions of eigenvectors first to obtain the proper stress components. We have

$$C^{I}\left(\xi \right)U^{\pm }=\left[\begin{array}{ccc} \frac{\mp 2\mu_{I}p_{I}\xi_{1}}{\sqrt{p_{I}^{2}+\xi^{2}}} & \frac{\mu_{I}\left(q_{I}^{2}-\xi^{2}\right)\xi_{1}}{\sqrt{q_{I}^{2}+\xi^{2}}\cdot \xi } & \pm \frac{\mu_{I}q_{I}\xi_{2}}{\xi }\\ \frac{\mp 2\mu_{I}p_{I}\xi_{2}}{\sqrt{p_{I}^{2}+\xi^{2}}} & \frac{\mu_{I}\left(q_{I}^{2}-\xi^{2}\right)\xi_{2}}{\sqrt{q_{I}^{2}+\xi^{2}}\cdot \xi } & \pm \frac{\mu_{I}q_{I}\xi_{1}}{\xi }\\ \frac{\mu_{I}\left(q_{I}^{2}-\xi^{2}\right)}{\sqrt{p_{I}^{2}+\xi^{2}}} & \pm \frac{2\mu_{I}q_{I}\xi^{2}}{\sqrt{q_{I}^{2}+\xi^{2}}\cdot \xi } & 0 \end{array}\right],$$ (3.18)

$$C^{\mathrm{II}}\left(\xi \right)U^{\pm }=\left[\begin{array}{ccc} \frac{\mp 2\mu_{\mathrm{II}}p_{\mathrm{II}}\xi_{1}}{\sqrt{p_{\mathrm{II}}^{2}+\xi^{2}}} & \frac{\mu_{\mathrm{II}}\left(q_{\mathrm{II}}^{2}-\xi^{2}\right)\xi_{1}}{\sqrt{q_{\mathrm{II}}^{2}+\xi^{2}}\cdot \xi } & \pm \frac{\mu_{\mathrm{II}}q_{\mathrm{II}}\xi_{2}}{\xi }\\ \frac{\mp 2\mu_{\mathrm{II}}p_{\mathrm{II}}\xi_{2}}{\sqrt{p_{\mathrm{II}}^{2}+\xi^{2}}} & \frac{\mu_{\mathrm{II}}\left(q_{\mathrm{II}}^{2}-\xi^{2}\right)\xi_{2}}{\sqrt{q_{\mathrm{II}}^{2}+\xi^{2}}\cdot \xi } & \pm \frac{\mu_{\mathrm{II}}q_{\mathrm{II}}\xi_{1}}{\xi }\\ \frac{\mu_{\mathrm{II}}\left(q_{\mathrm{II}}^{2}-\xi^{2}\right)}{\sqrt{p_{\mathrm{II}}^{2}+\xi^{2}}} & \pm \frac{2\mu_{\mathrm{II}}q_{\mathrm{II}}\xi^{2}}{\sqrt{q_{\mathrm{II}}^{2}+\xi^{2}}\cdot \xi } & 0 \end{array}\right].$$ (3.19)

Substituting the relevant matrices into Eqs. (2.39) and (2.48) we obtain the reflection coefficient matrices *R*+, *R*^−^, and R*^−^ respectively.

$$\begin{array}{c} R^{+}=\left[R_{ij}^{+}\right],\\ R_{11}^{+}=R_{22}^{+}=\left[{\left(q_{I}^{2}-\xi^{2}\right)}^{2}-4p_{I}q_{I}\xi^{2}\right]/\left[{\left(q_{I}^{2}-\xi^{2}\right)}^{2}+4p_{I}q_{I}\xi^{2}\right],\\ R_{12}^{+}=\left[4q_{I}\xi \left(q_{I}^{2}-\xi^{2}\right){\left(p_{I}^{2}+\xi^{2}\right)}^{1/2}\right]/\left\{{\left(q_{I}^{2}+\xi^{2}\right)}^{1/2}\left[{\left(q_{I}^{2}-\xi^{2}\right)}^{2}+4p_{I}q_{I}\xi^{2}\right]\right\},\\ R_{21}^{+}=-[4p_{I}\xi \left(q_{I}^{2}-\xi^{2}\right){\left(q_{I}^{2}+\xi^{2}\right)}^{1/2}]/\left\{{\left(p_{I}^{2}+\xi^{2}\right)}^{1/2}\left[{\left(q_{I}^{2}-\xi^{2}\right)}^{2}+4p_{I}q_{I}\xi^{2}\right]\right\},\\ R_{33}^{+}=-1,\\ R_{13}^{+}=R_{23}^{+}=R_{31}^{+}=R_{32}^{+}=0, \end{array}$$ (3.20)

$$\begin{array}{c} R^{-}=\left[R_{ij}^{+}\right],\\ R_{11}^{-}=\left[-\Delta_{1}^{+}\Delta_{2}^{-}+\Delta_{3}^{-}\Delta_{4}^{+}\right]/\Delta_{\mathrm{II}},\\ R_{12}^{-}=\left[-\Delta_{1}^{+}\Delta_{4}^{-}+\Delta_{4}^{+}\Delta_{1}^{-}\right]/\Delta_{\mathrm{II}},\\ R_{21}^{-}=\left[\Delta_{3}^{+}\Delta_{2}^{-}+\Delta_{2}^{+}\Delta_{3}^{-}\right]/\Delta_{\mathrm{II}},\\ R_{22}^{-}=\left[\Delta_{3}^{+}\Delta_{4}^{-}-\Delta_{2}^{+}\Delta_{1}^{-}\right]/\Delta_{\mathrm{II}},\\ R_{33}^{-}=\left(\mu_{I}q_{I}-\mu_{\mathrm{II}}q_{\mathrm{II}}\right)/\left(\mu_{I}q_{I}+\mu_{\mathrm{II}}q_{\mathrm{II}}\right),\\ R_{13}^{-}=R_{23}^{-}=R_{31}^{-}=R_{32}^{-}=0, \end{array}$$ (3.21)

where

$$\begin{array}{c} \Delta_{1}^{\pm }=\left\{\pm 2\mu_{1}q_{1}+\mu_{\mathrm{II}}\left[q_{2}\left(q_{2}^{2}+\xi^{2}\right)\mp q_{1}\left(2p_{2}q_{2}+\xi^{2}-q_{2}^{2}\right)\right]/\left(p_{2}q_{2}+\xi^{2}\right)\right\}/{\left(q_{1}^{2}+\xi^{2}\right)}^{1/2},\\ \Delta_{2}^{\pm }=\left\{\mp 2\mu_{1}p_{1}+\mu_{\mathrm{II}}\left[-p_{2}\left(q_{2}^{2}+\xi^{2}\right)\pm p_{1}\left(2p_{2}q_{2}+\xi^{2}-q_{2}^{2}\right)\right]/\left(p_{2}q_{2}+\xi^{2}\right)\right\}/{\left(p_{1}^{2}+\xi^{2}\right)}^{1/2},\\ \Delta_{3}^{\pm }=\left\{\mu_{I}\left(q_{1}^{2}-\xi^{2}\right)+\mu_{\mathrm{II}}\left[\left(2p_{2}q_{2}+\xi^{2}-q_{2}^{2}\right)\right]\pm p_{1}q_{2}(q_{2}^{2}+1)]/(p_{2}q_{2}+\xi^{2})\right\}/{\left(p_{1}^{2}+\xi^{2}\right)}^{1/2},\\ \Delta_{4}^{\pm }=\{\mu_{1}(q_{1}^{2}-\xi^{2})+\mu_{\mathrm{II}}[(\pm p_{2}q_{1}-1)(q_{2}^{2}+1)+(2p_{2}q_{2}+2\xi -q_{2}^{2})]/(p_{2}q_{2}+\xi^{2})\}/{\left(q_{2}^{2}+\xi^{2}\right)}^{1/2},\\ \Delta_{ΙΙ}=\Delta_{3}^{+}\Delta_{4}^{+}-\Delta_{1}^{+}\Delta_{2}^{+}, \end{array}$$

and

$$\begin{array}{c} R^{*-}=[R_{ij}^{*-}],\\ R_{11}^{*-}=[-\Delta_{1}^{*+}\Delta_{2}^{*-}+\Delta_{3}^{*-}\Delta_{4}^{*+}]/\Delta_{\mathrm{II}}^{*},\\ R_{12}^{*-}=(-\Delta_{1}^{*+}\Delta_{4}^{*-}+\Delta_{4}^{*+}\Delta_{1}^{*-})/\Delta_{\mathrm{II}}^{*},\\ R_{21}^{*-}=(\Delta_{3}^{*+}\Delta_{2}^{*-}+\Delta_{2}^{*+}\Delta_{3}^{*-})/\Delta_{\mathrm{II}}^{*},\\ R_{22}^{*-}=[\Delta_{3}^{*+}\Delta_{4}^{*-}-\Delta_{2}^{*+}\Delta_{1}^{*-}]/\Delta_{\mathrm{II}}^{*},\\ R_{33}^{*-}=(\mu_{I}q_{I}-\mu_{\mathrm{II}}q_{\mathrm{II}})/(\mu_{I}q_{I}+\mu_{\mathrm{II}}q_{\mathrm{II}}),\\ R_{13}^{*-}=R_{23}^{*-}=R_{31}^{*-}=R_{32}^{*-}=0, \end{array}$$ (3.22)

where

$$\begin{array}{c} \Delta_{1}^{*\pm }=\left\{\pm 2\mu_{1}q_{1}+\frac{\mu_{2}[{\left(q_{2}^{2}-1\right)}^{2}+4p_{2}q_{2}]}{p_{2}(q_{2}^{2}+1)}\right\}/{\left(q_{1}^{2}+1\right)}^{1/2},\\ \Delta_{2}^{*\pm }=\mp 2p_{1}\mu_{1}/{\left(p_{1}^{2}+1\right)}^{1/2},\\ \Delta_{3}^{*\pm }=\left\{\mu_{1}(q_{1}^{2}-1)\pm \frac{\mu_{2}p_{1}[{\left(q_{2}^{2}-1\right)}^{2}+4p_{2}q_{2}]}{p_{2}(q_{2}^{2}+1)}\right\}/{\left(p_{1}^{2}+1\right)}^{1/2},\\ \Delta_{4}^{*\pm }=\mu_{2}\frac{4q_{2}(p_{2}-q_{2})}{p_{2}(q_{2}^{2}+1)}+\frac{\rho_{2}\omega^{2}}{p_{2}}. \end{array}$$

In what follows let us first consider the interface condition for case 1, i.e., the welded interface. In the case of isotropic materials the inverse transform Eq. (2.43) takes the following form:

$${\left(G^{-}\right)}^{m\ell }=\sum_{k=1}^{\infty }{\left(-1\right)}^{k}[1+\delta_{k}^{\ell }[H(z-x_{3})-1]\frac{1}{4\pi^{2}}\sum_{\begin{array}{l} g=1,2,3\\ j=1,2,3 \end{array}}\oint_{\begin{array}{l} \left|\eta \right|=1\\ \phi =0 \end{array}}\frac{{\left(U^{-}\right)}_{j}^{m}R^{-}(j,g,k){\left[{\left(U^{{\left(-1\right)}^{k}}\right)}^{T}\right]}_{\ell }^{g}{\left(D^{{\left(-1\right)}^{k}}\right)}_{g}^{g}}{\frac{\partial \phi^{-}(\Omega ,\eta )}{\partial \Omega }}ds,$$ (3.29)

where *j* and *g* take 1, 2, and 3 and respectively represent the type of the final trip and the initial trip of the ray path to be a P ray, SV ray or SH ray in the layer, and

$$\phi^{-}(\Omega ,\eta )=-\Omega t+\eta_{\alpha }x_{\alpha }-2hk_{1}^{-}p_{I}-2hk_{2}^{-}q_{I},$$ (3.30)

where *k*^−^_1_ and *k*^−^_2_ are the numbers of times the layer is traversed by a P ray and S ray, respectively. They may take fractional values when the source or the detector is not on the top surface or the interface. Also,

$${\left(D^{-}\right)}_{k}^{\ell }=-{\left(2p_{I}\Omega b_{k}\right)}^{-1}\delta_{k}^{\ell },$$ (3.31)

where

$$b_{1}=a_{I}^{2}p_{I},\;b_{2}=b_{3}=c_{I}^{2}q_{I}.$$ (3.32)

**Fig. 2:**
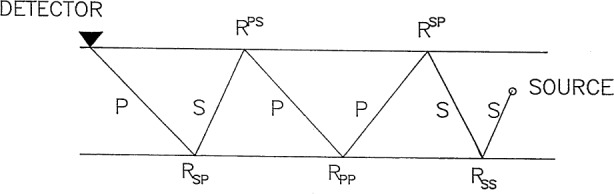
A typical ray path within the layer; p and s respectively represent the sections traveled with p wave speed and s wave speed. *R*^ps^, *R*_pp_, etc., respectively, represent the reflection coefficients of the top surface and the lower surface of the layer; the superscripts or subscripts represent the wave mode before and after reflection.

*R*^−^ (*j*, *g*, *k*) represents the sum of the products of all the reflection coefficients produced by the rays which travel from the source to the observation point (detector) and touch the top surface and the bottom surface of the layer. These rays have the same final trip *j* and the same initial trip *g* as well as the same total number of layer traverses *k* of the layer as shown in Fig. 2. I.e., they have the same arrival time. The concrete expression of *R*^−^ (*j*, *g*, *k*) will be discussed in the next section.

Similarly, we have

$${\left(G^{+}\right)}^{m\ell }=\sum_{k=1}^{\infty }{\left(-1\right)}^{k}\{1+\delta_{k}^{\ell }[H(x_{3}-z)-1]\}\frac{1}{4\pi^{2}}\sum_{\begin{array}{l} g=1,2,3\\ j=1,2,3 \end{array}}\oint_{\begin{array}{l} \left|\eta \right|=1\\ \phi =0 \end{array}}\frac{{\left(U^{+}\right)}_{j}^{m}R^{+}(j,g,k){\left[{\left(U^{{\left(-1\right)}^{k+1}}\right)}^{T}\right]}_{\ell }^{g}{\left(D^{{\left(-1\right)}^{k+1}}\right)}_{g}^{g}}{\frac{\partial \phi^{+}(\Omega ,\eta )}{\partial \Omega }}ds,$$ (3.33)

where

$$\phi^{+}(\Omega ,\eta )=-\Omega t+\eta_{\alpha }x_{\alpha }-2hk_{1}^{+}p_{I}-2hk_{2}^{+}q_{I},$$ (3.34)

$${\left(D^{+}\right)}_{k}^{\ell }=-{\left(2p_{I}\Omega b_{k}\right)}^{-1}\delta_{k}^{\ell }.$$ (3.35)

The meanings of the various quantities in Eqs. (3.33)–(3.35) are the same as before or are similar to that of the quantities in Eqs. (3.29)–(3.32).

Using

ϕ(Ω,η)=0, (3.36)

then Eqs. (3.30) and (3.34) can be written as

$${\frac{\partial \phi^{\pm }}{\partial \Omega }|}_{\phi =0}=\frac{1}{\Omega }\left(-\eta_{\alpha }x_{\alpha }-\frac{2hk_{1}^{\pm }}{p_{I}}-\frac{2hk_{2}^{\pm }}{q_{I}}\right).$$ (3.37)

### 5.1 Detector on the Top Surface

Usually, the detector (observation point) is put on the top surface of the layer; then Eqs. (3.29) and (3.33) can be further combined. If the detector was considered to be located an infinitesimal amount below the upper surface, we should have to take into account at almost the same instant the wave going upward before reflection and the reflected wave going downward which come from the same ray and have almost the same phase function. Substituting into Eqs. (3.29) and (3.33) with *k* = *n* + 1 and *k* = *n* + 2, respectively, and defining

$$\Gamma =U^{+}-U^{-}R^{+},$$ (3.38)

and substituting the previous relevant expressions into Eq. (3.38) we obtain

$$\Gamma =[\gamma_{ij}].$$ (3.39)

Here

$$\begin{array}{c} \gamma_{11}=[\eta_{1}4p_{I}q_{I}\left(q_{I}^{2}+1\right)]/{\left[p_{I}^{2}+1\right]}^{1/2}\Delta_{I},\\ \gamma_{12}=[-\eta_{1}2q_{I}\left(q_{I}^{2}-1\right){\left(q_{I}^{2}+1\right)}^{1/2}]/\Delta_{I},\\ \gamma_{13}=-2\eta_{2},\\ \gamma_{21}=[\eta_{2}4p_{I}q_{I}\left(q_{I}^{2}+1\right)]/{\left[p_{I}^{2}+1\right]}^{1/2}\Delta_{I},\\ \gamma_{22}=[-\eta_{2}2q_{I}\left(q_{I}^{2}-1\right){\left(q_{I}^{2}+1\right)}^{1/2}]/\Delta_{I},\\ \gamma_{23}=2\eta_{1},\\ \gamma_{31}=[-2p_{I}\left(q_{I}^{2}-1\right)\left(q_{I}^{2}+1\right)]/{\left[p_{I}^{2}+1\right]}^{1/2}\Delta_{I},\\ \gamma_{32}=[-4p_{I}q_{I}{\left(q_{I}^{2}+1\right)}^{1/2}]/\Delta_{I},\\ \gamma_{33}=0,\\ \Delta_{I}={\left(q_{I}^{2}-1\right)}^{2}+4p_{I}q_{I}. \end{array}$$

Then Eqs. (3.29) and (3.33) can be combined and written as one formula:

$$G^{m\ell }=\sum_{n=0}^{\infty }\sum_{\begin{array}{l} g=1,2,3\\ j=1,2,3 \end{array}}\oint_{\begin{array}{l} \left|\eta \right|=1\\ \phi =0 \end{array}}{\left(-1\right)}^{n}\gamma_{j}^{m}R(j,g,n){\left(U^{{\left(-1\right)}^{n}}\right)}_{g}^{\ell }D\phi (k_{1},k_{2})P(g)ds,$$ (3.40)

where

$$\phi =-\tau y+d-k_{1}p_{I}-k_{2}q_{I},$$ (3.41)

$$\begin{array}{l} \tau =c_{I}t/2h,\;y=\Omega /c_{I}\;\xi_{\alpha }=x_{\alpha }/2h,\;d=\xi_{\alpha }\eta_{\alpha },\\ p_{I}={\left(\alpha_{1}^{2}y^{2}-1\right)}^{1/2},\;q_{I}={\left(y^{2}-1\right)}^{1/2},\;\varsigma =z/2h,\\ \alpha_{1}=c_{I}/a_{I},\;k_{\beta }=n_{\beta }+\delta_{\beta }^{g}\left(\frac{1}{2}-\varsigma \right),\;\beta =1,2. \end{array}\}$$ (3.42)

$$D\phi \left(k_{1},k_{2}\right)=\frac{1}{16\pi^{2}\rho_{I}h\left(d+\frac{k_{1}}{p_{I}}+\frac{k_{2}}{q_{I}}\right)},$$ (3.43)

$$P(g)=\frac{1}{b_{g}}.$$ (3.44)

### 5.2 Source and Detector on the Top Surface

Similarly, when the source is also located on the top surface of the layer then Eq. (3.40) may be written as

$$G^{m\ell }=\sum_{n=0}^{\infty }\sum_{\begin{array}{l} g=1,2,3\\ j=1,2,3 \end{array}}\oint_{\begin{array}{l} \left|\eta \right|=1\\ \phi =0 \end{array}}{\left(-1\right)}^{n}\gamma_{j}^{m}R(j,g,n){\left(\psi \right)}_{g}^{l}D\phi (k_{1},k_{2})P(g)ds,$$ (3.45)

where

$$\phi =-\tau y+d-k_{1}p_{I}-k_{2}q_{I}.$$ (3.46)

${\left(\psi \right)}_{g}^{l}$ is a component of the following matrix,

$$\psi =R^{+}{\left(U^{+}\right)}^{T}-{\left(U^{-}\right)}^{T}.$$ (3.47)

Obviously, substituting the corresponding quantities with “*” into Eqs. (3.29), (3.33), (3.40), and (3.45) gives the formulas corresponding to case 2, i.e., the liquid coupled interface.

If we are only interested in the early time or short time arrival of the signal received by the detector, only the finite terms of all the previous integrals need be computed, because the reflected rays with more reflections do not have enough time to arrive at the detector.

Following Ref. [3], we introduce the following transform

$$\begin{array}{l} \eta_{1}=\cos\theta =d/x,\\ \eta_{2}=\sin\theta =\pm {\left(x^{2}-d^{2}\right)}^{1/2}/x\\ ds=d\theta \\ y(\theta )=y(-\theta ) \end{array}\}$$ (3.48)

where *x* = |*x*| and *θ* is the angle measured around |η| = 1 from the direction of the vector *x*. Note that if *y* is a root of

$$-\tau y+d-k_{1}i{\left(1-\alpha_{1}^{2}y^{2}\right)}^{1/2}-k_{2}i{\left(1-y^{2}\right)}^{1/2}=0,$$ (3.49)

for a given value of *d*, then −*y* will be a root of the following equation when *d* is replaced by −*d*:

$$\tau y-d-k_{1}i{\left(1-\alpha_{1}^{2}y^{2}\right)}^{1/2}-k_{2}i{\left(1-y^{2}\right)}^{1/2}=0.$$ (3.50)

Thus, if *d* and *y* form a solution to Eq. (3.49) or (3.50), then $\tilde{d}$, and $\tilde{y}$ (they are the complex conjugates of *d*, *y*) are solutions to Eqs. (3.50) or (3.49), while $-\tilde{d}$, and $-\tilde{y}$ are solutions to Eqs. (3.49) or (3.50). When all the branch cuts for the square root functions in the above equations were taken along the real axis between each pair of branch points, using the above relations Eqs. (3.48)–(3.50) and, finally, the alternate form *ϕ* = *ε*i for the integration locus, then every term of the integrals Eqs. (3.40) and (3.45) can be simplified to the form

$$2\int_{-x-i0}^{x-i0}I[y(d),d]\frac{dd}{{\left(x^{2}-d^{2}\right)}^{1/2}},$$ (3.51)

where the integrand *I*[*y*(*d*), *d*] represents the integrand of each term, including all the factors of Eq. (3.40) or (3.45). The denominator (*x*^2^ − *d*^2^)^1/2^ and the constant factor 2 are introduced by the variable transform Eq. (3.48) and the change of the integration path. All the singularities of the integrand of Eq. (3.51) are located in the upper half or on the real axis of the *d*-plane. For a detailed derivation of Eq. (3.51), refer to Ref. [3].

## 6. Automatic Generation of Ray Paths and of Corresponding Products of Reflection Coefficients

**Fig. 3:**
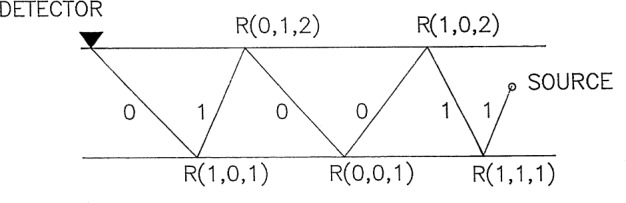
A typical ray path within the layer; p and s respectively are replaced by 0 and 1; *R*^ps^, etc., by *R*(0,1,2), etc.

**Fig. 4:**
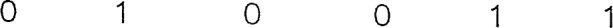
An example of a binary sequence, that uniquely characterizes the ray path in Fig. 3.

In general, there are many rays which arrive at the detector at the same time owing to the multiple reflection and the mode conversion of the incident P ray or SV ray emitted from the force source in the layer, and therefore it is not necessary to separately compute each term in the integrals mentioned above. The question of how many rays will arrive at the detector at the same time for a given configuration is a problem of combinatorics. Although explicit formulae for summing up the rays with the same arrival time to form a single integral can be derived for the case of a plate because the reflection coefficients at the top surface and bottom surface are the same [3,12], similar formulae are not easily derived for the present case of a layer on a substrate. Even though these formulae can be obtained, the actual program would also be rather complicated to carry out for a summation with reflection coefficients at the top surface which are different from those at the lower surface. In the following we will give a special counting scheme for this problem. First, let us change the notation in Fig. 2 and we obtain Fig. 3. Notation “1” and “0” in Fig. 3 respectively corresponds to notation “s” and “p” in Fig. 2. As a result it is not difficult to understand the meaning of R(0,1,1), R(1,1,2), etc. The first argument denotes the type of incident ray while the second one the reflected ray. The third arguments, “1” and “2”, respectively, represent the lower surface and the top surface of the layer. Obviously, the configuration of the rays in Fig. 3 may be expressed in a sequence consisting of “0” and “1” as shown in Fig. 4. On the other hand, the sequence in Fig. 4 may also be considered as a binary notation of some integer.

To find how many rays travel through the thickness of the layer three times with p-wave speed and three times with s-wave speed, therefore, is the same problem as to find how many different six-bit binary integers can be constructed by three 1’s and three 0’s. Using a bit-test program it is easy to get the count and find all those binary numbers that correspond to all the ray paths with the proper p-wave and s-wave sequences. An outline of the procedures for assembling the proper sequence of the reflection coefficients for each ray path is given in the following for the case of the source and the detector located on the top surface of the layer.

1. For given *k*_1_ and *k*_2_, the number of layer traverses with p-wave speed and s-wave speed respectively, there is in general more than one ray path which will have the same arrival time. But each ray may have a different reflection sequence. From Figs. 3 and 4, the sequence for each ray path has a corresponding N-bit binary number representation of *k*_1_ 1’s and *k*_2_ 0’s; *N* = *k*_1_ + *k*_2_.
2. Using the bit-test program, we can determine IP, the number of distinct binary sequence with *k*_1_ 1’s and *k*_2_ 0’s, store all such *N*-bit binary numbers in an array, IA, of dimension IP. Each binary number will have *k*_1_ 1’s and *k*_2_ 0’s, and they are all different.
3. For each binary number in IA we can construct a product of the reflection coefficients according to Fig. 3.
4. Summing all the products thus obtained gives all the rays which arrive at the detector at the same time.

The procedure outlined above is rather efficient in terms of computation speed as long as *N* is limited to a small number, say less than 16. The results in the case of a plate without the lower half space have been checked against the results computed using explicit formulae previously derived [12]. These same formulae for a plate cannot be used for the case of a layer on a half-space.

## 7. Singularities and Wave Front Arrivals

The integrand of Eqs. (3.40) or (3.45) may contain singularities, and they can be divided into three categories that respectively correspond to three different types of wave front arrivals: 1) The body waves relevant to the regular reflected rays which are determined from the singularities of *∂*ϕ/∂*y* = 0, 2) The interface waves, including Rayleigh surface waves, Stoneley interface waves, and the other possible leaky waves, which are determined from the singularities of Δ_I_ = 0 or Δ_II_ = 0 in the denominators of the reflection coefficients, and 3) The head waves determined from the singularities of the branch points of the square root functions.

Following the analysis in Ref. [3] we will show that all the above mentioned singularities of the integrands in the *y*-plane will be located in the upper half or on the real axis of the *y*-plane and their mapping onto the *d*-plane, which is determined from Eq. (3.48), will locate them in the upper half or on the real axis of the *d*-plane. So the integration path will never touch the singularities of the *d*-plane when we use formula (3.51) to compute the Green’s functions and choose the integration path slightly below the real axis. This is indeed a main advantage of the Willis method.

Let us first consider the head wave arrivals which are related to the singularities of the branch points.

## 8. Head Waves

Case 1. Welded interface.

In this case we have the following four square root functions:

$$\begin{array}{l} p_{I}={\left(\alpha_{1}^{2}y^{2}-1\right)}^{1/2},\;q_{I}={\left(\alpha_{2}^{2}y^{2}-1\right)}^{1/2},\\ P_{\mathrm{II}}={\left(\alpha_{3}^{2}y^{2}-1\right)}^{1/2},\;q_{\mathrm{II}}={\left(\alpha_{4}^{2}y^{2}-1\right)}^{1/2}, \end{array}$$ (4.1)

where

$$\alpha_{1}=c_{I}/a_{I},\;\alpha_{2}=1,\;\alpha_{3}=c_{I}/a_{\mathrm{II}},\;\alpha_{4}=c_{I}/c_{\mathrm{II}}.$$ (4.2)

Their branch points are respectively

$$y_{1}=\pm \alpha_{1}^{-1},\;y_{2}=\pm 1,\;y_{3}=\pm \alpha_{3}^{-1},\;y_{4}=\pm \alpha_{4}^{-1}.$$ (4.3)

For convenience of discussion, without losing generality we assume the source and detector are located on the top surface of the layer and

$$a_{\mathrm{II}}>c_{\mathrm{II}}>a_{I}>c_{I}.$$ (4.4)

Then from Eq. (4.2) we have

$$\alpha_{3}<\alpha_{4}<\alpha_{1}<1.$$ (4.5)

The relation between *y* and *d* satisfies the equation

$$d=\tau y+k_{1}i{\left(1-\alpha_{1}^{2}y^{2}\right)}^{1/2}+k_{2}i{\left(1-y^{2}\right)}^{1/2},$$ (4.6)

where

$$\tau =tc_{1}/2h,\;d=x_{\alpha }\eta_{\alpha }/2h.$$ (4.7)

A simple analysis of Eq. (4.6) shows that when all the branch cuts were taken on the real axis of the *y*-plane between each pair of branch points, the mapping of the branch cuts in the *d*-plane will be located in the upper half and on the real axis of the *d*-plane.

The following gives some examples of head wave arrivals corresponding to the branch points.

**Fig. 5:**
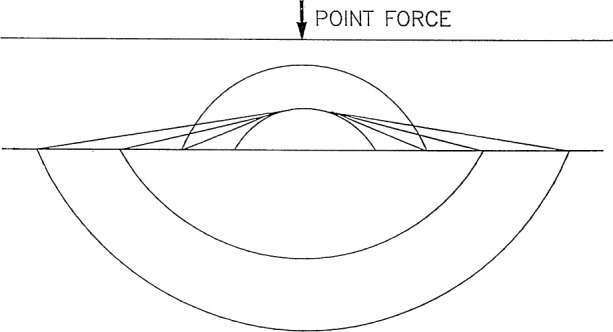
Schematic representation of the head waves in the layer generated by reflection and refraction of a spherical P wave and a spherical S wave impinging on the interface.

1. The head wave arrivals corresponding to branch point *y*_1_.
  From Eq. (4.3) we have
  $$y_{1}=1/\alpha_{1}=a_{I}/c_{I},$$ (4.8)
  and
  y=cscθ, (4.9)
  where *θ* is the incident angle. Substituting Eq. (4.9) into Eq. (4.8) and writing *θ* as *θ_α_*_1_ we obtain
  $$\sin\theta_{\alpha 1}=c_{I}/a_{I}.$$ (4.10)
  Equation (4.10) is the critical condition satisfied by the critical angle *θ_α_*_1_ which leads to the generation of one type of head wave. The head waves of this type is named SP*S head waves. Here S and P respectively denote an SV ray and P ray in the layer. For a detailed physical mechanisms of the generation of head waves, refer to Refs. [6] and [7].
  Substituting Eq. (4.8) into Eq. (4.6) and letting *k*_1_ = 0 we obtain the normalized arrival time of the SP*S head waves,
  $$\tau =d\frac{c_{I}}{a_{I}}+k_{2}\frac{c_{I}}{a_{I}}{\left[{\left(\frac{a_{I}}{c_{I}}\right)}^{2}-1\right]}^{1/2}.$$ (4.11)
  For example, when *k*_2_ = 2 it is just the arrival time of the SP*S head wave, where SP*S also represents the propagation path of the head wave ray. It consists of three sections, the first section of the notation, S, represents a critically incident S ray in the layer, the second, P*, represents a P ray propagating along the interface but on the side of the layer, and the final S is a critically reflected S ray in the layer. Later on, the lower case letter p or s with a star will represent a P ray or S ray propagating along the interface on the side of the half-space.
2. The wave arrival corresponding to branch point *y*_2_.
  From Eq. (4.3) we have
  $$y_{2}=1.$$ (4.12)
  The corresponding critical angle satisfies
  $$\sin\theta_{\alpha 2}=1,\text{or}\;\theta_{\alpha 2}=\pi /2;$$ (4.13)
  this condition seems as if the S ray emitted from the source propagates along the top surface of the layer. We denote this ray by S*. It is possible only if *k*_1_ = 0 and *k*_2_ = 0. Letting *y*_2_ = 1 in Eq. (4.6), we obtain the normalized arrival time of this ray
  τ=d. (4.14)
3. The head wave arrivals corresponding to branch point *y*_3_.
  From Eq. (4.3) we have
  $$y_{3}=a_{\mathrm{II}}/c_{I}.$$ (4.15)
  The corresponding critical angle is
  $$\theta_{\alpha 3}=\sin^{-1}(c_{I}/a_{\mathrm{II}}).$$ (4.16)
  In this case we may have two kinds of head waves that are excited by the same critically incident S ray but have different critically reflected S and P rays. With *k*_1_ = 0 and using Eq. (4.15) in Eq. (4.6) we obtain the normalized arrival time of one kind of head wave
  $$\tau =d\frac{c_{I}}{a_{\mathrm{II}}}+k_{2}\frac{c_{I}}{a_{\mathrm{II}}}{\left[{\left(\frac{a_{\mathrm{II}}}{c_{I}}\right)}^{2}-1\right]}^{1/2}.$$ (4.17)
  When *k*_2_ = 2 we obtain the arrival time of the Sp*S head wave.
  Using Eq. (4.15) in Eq. (4.6) we obtain the normalized arrival time of the other kind of head wave
  $$\tau =d\frac{c_{I}}{a_{\mathrm{II}}}+k_{I}\frac{c_{I}}{a_{\mathrm{II}}}{\left[{\left(\frac{a_{\mathrm{II}}}{a_{I}}\right)}^{2}-1\right]}^{1/2}+k_{2}\frac{c_{I}}{a_{\mathrm{II}}}{\left[{\left(\frac{a_{\mathrm{II}}}{c_{I}}\right)}^{2}-1\right]}^{1/2}.$$ (4.18)
  When *k*_1_ = 1 and *k*_2_ = 1, it is the arrival time of the Sp*P head wave.
4. The head wave arrivals corresponding to branch point *y*_4_.
  From Eq. (4.3) we have
  $$y_{4}=c_{\mathrm{II}}/c_{I}.$$ (4.19)
  The corresponding critical angle is
  $$\theta_{\alpha 4}=\sin^{-1}\left(c_{I}/c_{\mathrm{II}}\right).$$ (4.20)
  In this case we also have two kinds of head waves. With *k*_1_ = 0 and using Eq. (4.19) in Eq. (4.6) we obtain the normalized arrival time of one kind of head wave
  $$\tau =d\frac{c_{I}}{a_{\mathrm{II}}}+k_{2}\frac{c_{I}}{c_{\mathrm{II}}}{\left[{\left(\frac{c_{\mathrm{II}}}{c_{I}}\right)}^{2}-1\right]}^{1/2}.$$ (4.21)
  When *k*_2_ = 2 it is the arrival time of the Ss*S head wave.
  Using Eq. (4.19) in Eq. (4.6), we obtain the normalized arrival time of the other kind of head wave
  $$\tau =d\frac{c_{I}}{c_{\mathrm{II}}}+k_{1}\frac{c_{I}}{c_{\mathrm{II}}}{\left[{\left(\frac{c_{\mathrm{II}}}{a_{I}}\right)}^{2}-1\right]}^{1/2}+k_{2}\frac{c_{I}}{c_{\mathrm{II}}}{\left[{\left(\frac{c_{\mathrm{II}}}{c_{I}}\right)}^{2}-1\right]}^{1/2}.$$ (4.22)
  When *k*_1_ = 1 and *k*_2_ = 1, it is the arrival time of the Ss*P head wave.
  It may be seen that the branch points *y*_1_, *y*_3_, and *y*_4_ correspond to the arrivals of various head wave rays, while the branch point *y*_2_ seems to correspond to the arrival of the S ray propagating along the top surface of the layer. These head waves all are excited by the S ray emitted from the source under the conditions of Eq. (4.4). A picture of the wave fronts of the head waves in the layer generated by reflection and refraction of a spherical P wave and a spherical S wave impinging on the interface is shown in Fig. 5.
  In order to describe the arrivals of various head wave rays and the P ray propagating along the top surface of the layer excited by the P ray emitted from the source, we need to change the form of the relations mentioned above. We write
  $$y=\frac{\Omega }{c_{I}}=\frac{a_{I}\Omega }{a_{I}c_{I}}=\frac{a_{I}\Omega }{c_{I}a_{I}}=\frac{a_{I}}{c_{I}}y_{p},$$ (4.23)
  where
  $$y_{p}=\Omega /a_{I}.$$ (4.24)
  Substituting Eq. (4.23) into Eq. (4.1) gives
  $$\begin{array}{l} p_{I}={\left(\beta_{1}^{2}y_{p}^{2}-1\right)}^{1/2},\;q_{I}={\left(\beta_{2}^{2}y_{p}^{2}-1\right)}^{1/2},\\ p_{\mathrm{II}}={\left(\beta_{3}^{2}y_{p}^{2}-1\right)}^{1/2},\;q_{\mathrm{II}}={\left(\beta_{4}^{2}y_{p}^{2}-1\right)}^{1/2}, \end{array}$$ (4.25)
  where
  $$\beta_{1}=1,\;\beta_{2}=a_{I}/c_{I},\;\beta_{3}=a_{I}/a_{\mathrm{II}},\;\beta_{4}=a_{I}/c_{\mathrm{II}}.$$ (4.26)
  Substituting Eq. (4.23) into Eq. (4.6) we obtain
  $$d=\tau \frac{a_{I}}{c_{I}}y_{p}+k_{1}i{\left(1-y_{p}^{2}\right)}^{1/2}+k_{2}i{\left[1-{\left(\frac{a_{I}}{c_{I}}y_{p}\right)}^{2}\right]}^{1/2}.$$ (4.27)
  Obviously, the transform of Eq. (4.23) maps the original *y*-plane onto the *y*_p_-plane, whereas the original branch points of the *y*-plane become those of the *y*_p_-plane as follows:
  $$y_{p1}=\pm 1,\;y_{p2}=\pm \beta_{2}^{-1},\;y_{p3}=\pm \beta_{3}^{-1},\;y_{p4}=\pm \beta_{4}^{-1},$$ (4.28)
  Similarly, we may discuss the arrivals corresponding to the branch points of Eq. (4.28).
5. The head wave arrivals corresponding to the SH ray.

Since the SH rays do not produce mode conversion and their speed is equal to that of the SV rays, the arrivals of the head waves excited by the incident SH rays emitted from the source are the same as those of the Ss*S head waves. These head waves may be called the Hh*H head waves.

Case 2. Liquid coupled interface.

In this case the number and the distribution of the branch points are the same as those in the case of the welded interface. The arrival time of various head waves and surface P and S rays are the same as those of the welded interface. (The polarities and amplitudes have important diagnostic differences; see Computed Results and Discussion.)

## 9. Interface Waves

The interface waves include the Stoneley waves and the leaky waves which propagate along the interface of two different solid media [8]. When one of the media is vacuum we obtain the Rayleigh waves propagating along the free surface of the solid. The Stoneley waves do not attenuate and the leaky waves attenuate when propagating along the interface. Unlike the Rayleigh waves, the Stoneley waves can exist only if some parameters of the media satisfy certain conditions [9]. The singularities corresponding to the Stoneley waves come from the real roots of the so called Stoneley equation, i.e., ***Δ***_II_ = 0 on the top Riemman sheet of the *y*-plane, while the singularities corresponding to the leaky waves come from the complex roots of the Stoneley equation on the other Riemman sheets of the *y*-plane and therefore have some attenuation [10].

Suppose the source and the detector are located on the top surface; in this case it can be shown from Eq. (3.49) that the mapping of the real root singularities corresponding to the Stoneley waves onto the *d*-plane will locate them in the upper half of the *d*-plane, while the mapping of the complex root singularities corresponding to the leaky waves onto the *d*-plane will never occur on the top Riemman sheet of the *d*-plane. Thus only the roots of the Rayleigh equation ***Δ***_I_ = 0 will be considered. Letting the denominator of the reflection coefficients of the top surface be equal to zero, we obtain the following Rayleigh equation:

$${\left(q_{1}^{2}-1\right)}^{2}+4p_{I}q_{I}=0.$$ (4.29)

Using the principle of the argument, it can be proved that Eq. (4.29) has only two real roots, +*y*_R_ and −*y*_R_. Obviously, only the positive real root is of interest. The speed of the Rayleigh waves is less than that of the S waves, and so *y*_R_ < 1.

Substituting *y*_R_ into Eq. (4.6) and letting *k*_1_ = 0 and *k*_2_ = 0, because the source and the detector are located on the top surface, we have

$$d=\tau y_{R}.$$ (4.30)

Since *τ* and *y*_R_ are real numbers, the *d* corresponding to the singularity *y*_R_ should be located on the real axis of the *d*-plane. The arrival time of the Rayleigh waves is determined from Eq. (4.30) if *d* and *y*_R_ are known.

## 10. Regular Reflected Rays

Analysis shows that the singularities of ∂*ϕ*/∂*y* = 0 which relate to the regular reflected rays are located on the real axis of the *y*-plane and so the mapping of these singularities onto the *d*-plane will locate them on the real axis of the *d*-plane from Eq. (3.49). It is not difficult to understand this consequence if we note that *y* = csc*θ*, where *θ* is the incident angle of the ray, and *d* is the wave front distance satisfying Snell’s law. When *d*, *α*_1_, *k*_1_, and *k*_2_ are given, we have a group of specified rays that have the same arrival time *τ* satisfying Eq. (4.6) and the following equation:

$$d=\frac{k_{1}}{{\left(\alpha_{1}^{2}y^{2}-1\right)}^{1/2}}+\frac{k_{2}}{{\left(y^{2}-1\right)}^{1/2}}.$$ (4.31)

Equation (4.31) may be obtained using Snell’s law. Obviously, we must first know the value of *y* in order to get the arrival time *τ* It is seen from Eqs. (4.6) and (4.31) that this is a problem of solving a nonlinear algebraic equation in *y*. Following Ref. [12], the solution for *y* is obtained using a simple, intuitive, iterative technique. The arrival time *τ* is determined by substituting the solution obtained into Eq. (4.6).

## 11. Integration Technique and Computation Procedures

The previous analysis shows that when we use Eq. (3.51) and choose the path of integration below the real axis of the *d*-plane, it will not touch the singularities of the integrand, which include Rayleigh poles and all the branch points of the square root functions. When all the branch cuts are taken along the real axis of the *y*-plane, then their mapping in the *d*-plane will locate them in the upper half-plane and on the real axis. This is indeed a main advantage of the Willis method.

The actual procedure for numerically computing the Green’s tensor can be summarized as follows:

1. For given material properties and test configuration represented by *x*, the distance between source and detector, a time of arrival table is computed first. The arrivals include all the regular reflected rays and all possible head waves and Rayleigh wave; each has an associated pair of number *k*_1_ and *k*_2_, denoting the group of the ray paths. The arrivals are sorted according to successive time sequences.
2. To compute a particular component of the Green’s tensor for a specific time, *t*, the number of possible arrivals, *N*, can be determined by a comparison with the time of arrival table computed in step 1. *N* is also the number of terms in Eq. (3.45) that need to be computed; each term consists of one definite integral.
3. For each integral to be numerically computed, the integrand consists of IP terms and each is a product of a unique sequence of reflection coefficients and some other factors. Both IP and the sequence series are computed by calling a bit test program as explained in Sec. 6.
4. The numerical integration is done by providing a function that computes the integrand for a given integration variable, *d*, along with the integration limits. The function is computed first by solving the equation *ϕ* = 0 for *y* for a given *d*, then computing the IP terms one at a time and summing; each term has a factor which corresponds to the product of all the sequences of reflection coefficients mapped by a binary number. A numerical integration subroutine is applied which handles integrands with removable singularities particularly well and also provides error estimates.
5. The current program was tested by considering the case when the lower half-space is a vacuum; i.e., the case when the structure is a plate. Results obtained are identical to the results obtained by the program to compute the Green’s tensor of a plate developed previously [12] which had also been checked experimentally.

## 12. Computed Results and Discussion

A FORTRAN program has been developed to numerically compute the Green’s tensor for a layer on a half-space with three different bonding conditions between the layer and the half-space. The program is written in such a way that for given isotropic material parameters, maximum observation time, subscript of the component of the Green’s tensor, number of sampling points, and distance between the source and detector, the program will compute the displacement at each sampling time. Furthermore, the arrivals of various rays are also computed and identified. The current limitation of the program is that the positions of the source and detector must be located on the top surface of the layer. However, the program will be modified to be applicable to the case of the source and detector located at arbitrary positions in the layer.

**Fig. 6:**
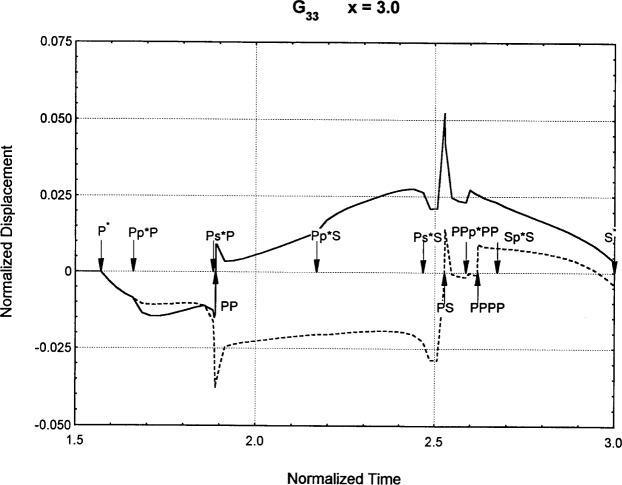
Green’s function ***G***_33_. *X* = 3. (A) The dashed curve is the result for a liquid coupled interface. (B) The solid curve is the result for a welded interface.

**Fig. 7:**
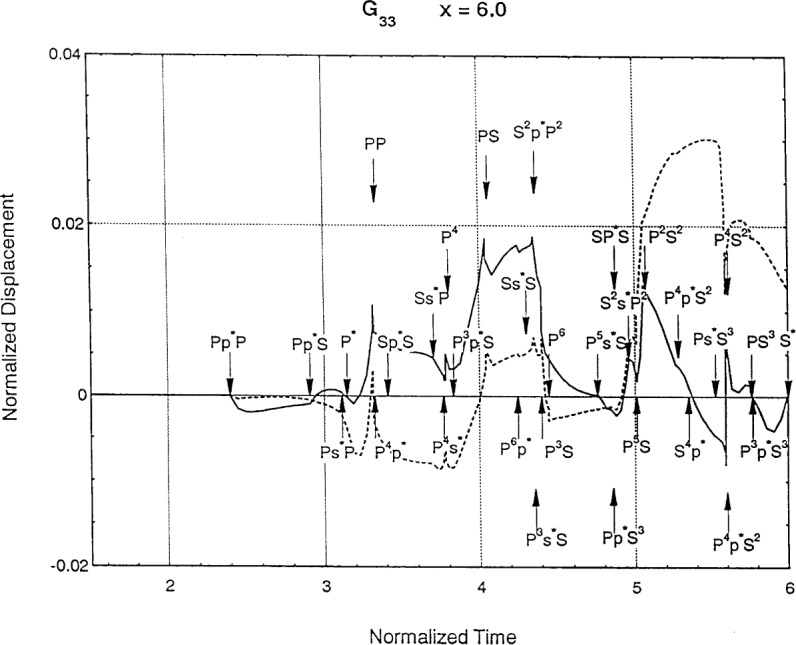
Green’s function ***G***_33_. *X* = 6. (A) The dashed curve is the result for a liquid coupled interface. (B) The solid curve is the result for a welded interface.

**Fig. 8:**
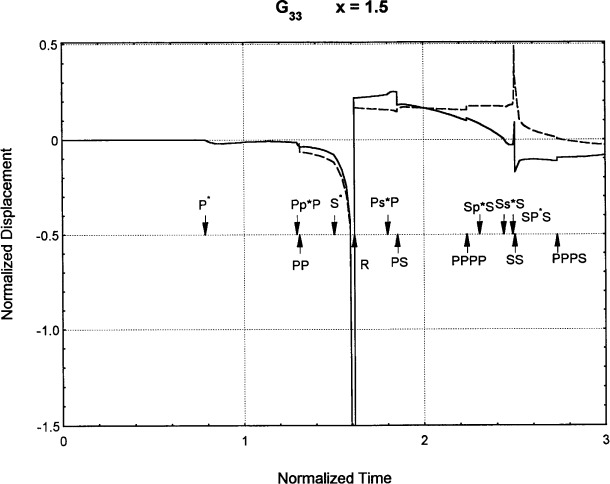
Green’s function ***G***_33_. *X* = 1.5. (A) The dashed curve is the result for a liquid coupled interface. (B) The solid curve is the result for a welded interface.

**Fig. 9:**
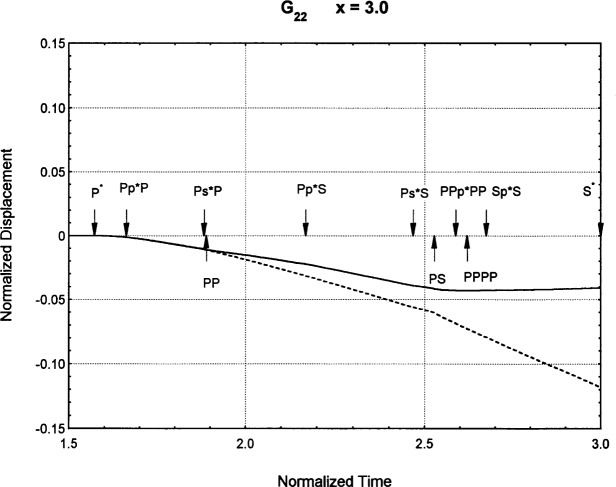
Green’s function ***G***_22_. *X* = 3. (A) The dashed curve is the result for a liquid coupled interface. (B) The solid curve is the result for a welded interface.

**Fig. 10:**
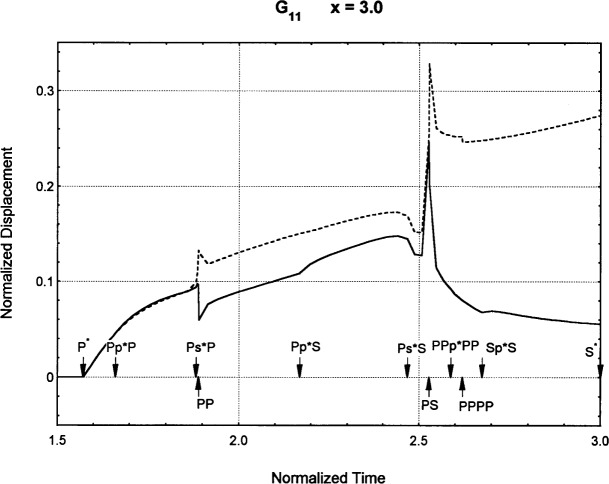
Green’s function ***G***_11_. *X* = 3. (A) The dashed curve is the result for a liquid coupled interface. (B) The solid curve is the result for a welded interface.

**Fig. 11:**
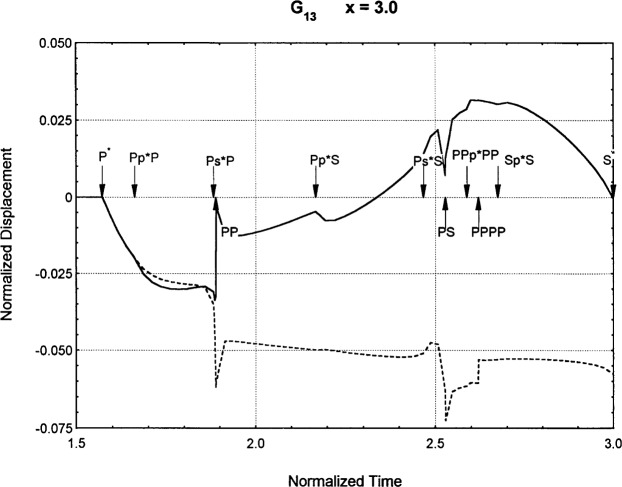
Green’s function ***G***_13_. *X* = 3. (A) The dashed curve is the result for a liquid coupled interface. (B) The solid curve is the result for a welded interface.

**Fig. 12:**
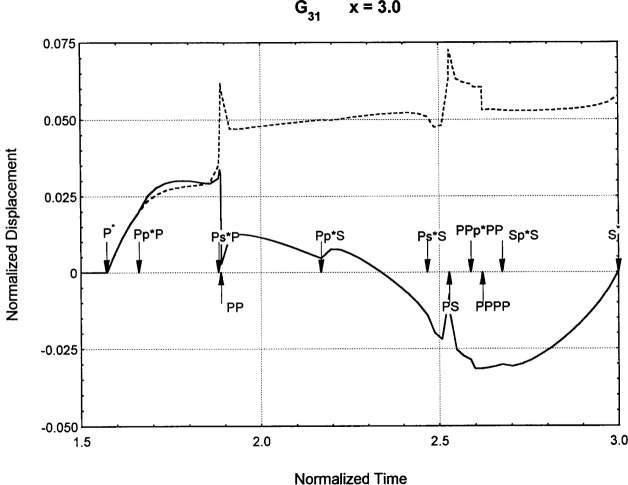
Green’s function ***G***_31_. *X* = 3. (A) The dashed curve is the result for a liquid coupled interface. (B) The solid curve is the result for a welded interface.

**Fig. 13:**
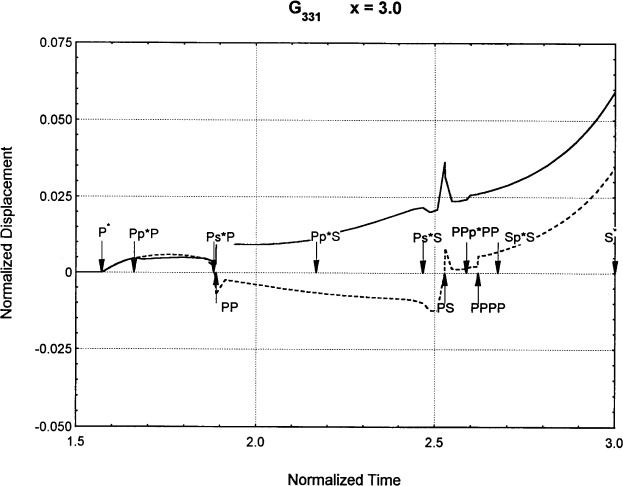
The spatial derivative of the Green’s function ***G***_331_. *X* = 3. (A) The dashed curve is the result for a liquid coupled interface. (B) The solid curve is the result for a welded interface.

**Fig. 14:**
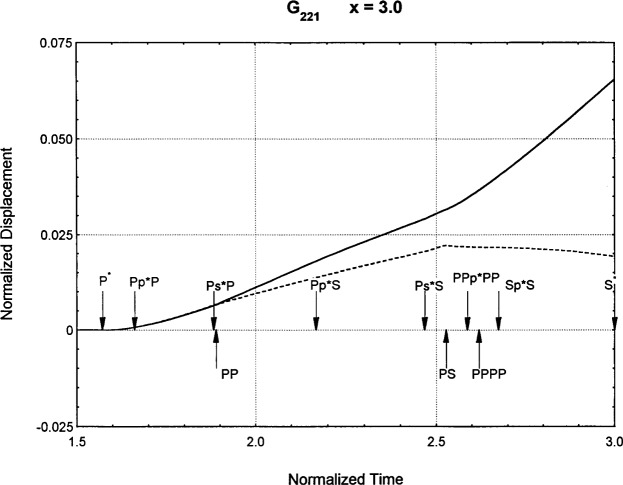
The spatial derivative of the Green’s function ***G***_221_. *X* = 3. (A) The dashed curve is the result for a liquid coupled interface. (B) The solid curve is the result for a welded interface.

**Fig. 15:**
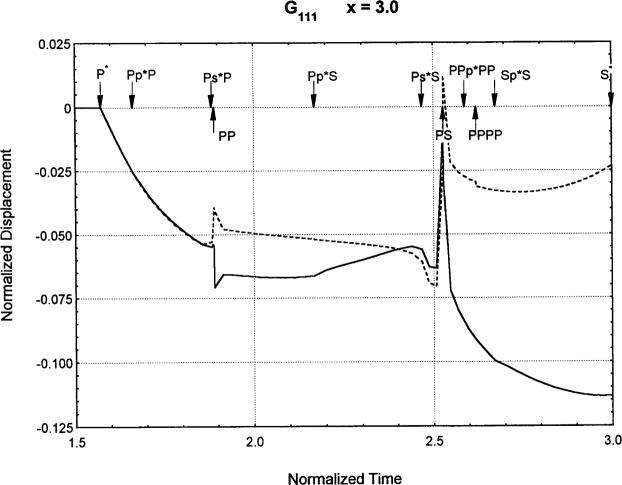
The spatial derivative of the Green’s function ***G***_111_. *X* = 3. (A) The dashed curve is the result for a liquid coupled interface. (B) The solid curve is the result for a welded interface.

**Fig. 16:**
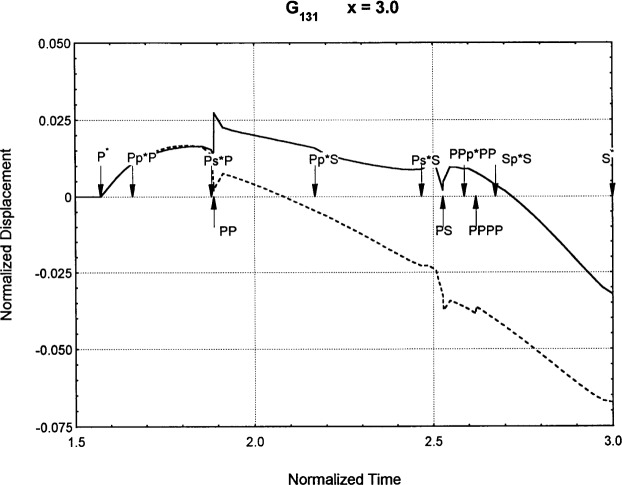
The spatial derivative of the Green’s function ***G***_131_. *X* = 3. (A) The dashed curve is the result for a liquid coupled interface. (B) The solid curve is the result for a welded interface.

**Fig. 17:**
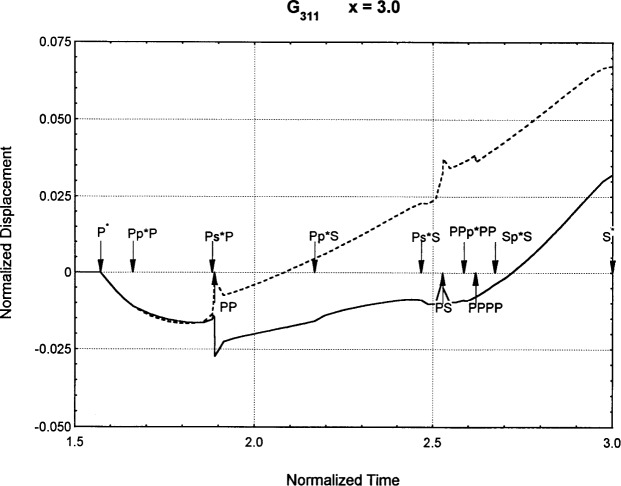
The spatial derivative of the Green’s function ***G***_311_. *X* = 3. (A) The dashed curve is the result for a liquid coupled interface. (B) The solid curve is the result for a welded interface.

**Fig. 18:**
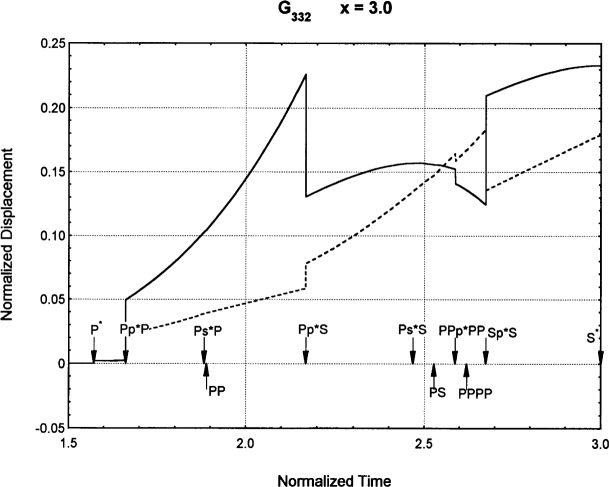
The spatial derivative of the Green’s function ***G***_332_. *X* = 3. (A) The dashed curve is the result for a liquid coupled interface. (B) The solid curve is the result for a welded interface.

**Fig. 19:**
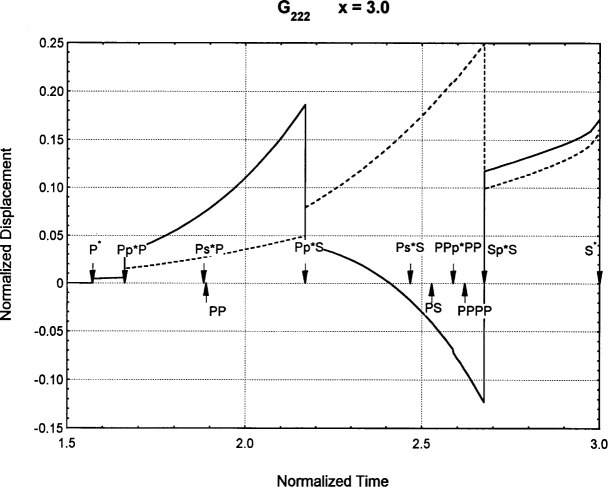
The spatial derivative of the Green’s function ***G***_222_. *X* = 3. (A) The dashed curve is the result for a liquid coupled interface. (B) The solid curve is the result for a welded interface.

**Fig. 20:**
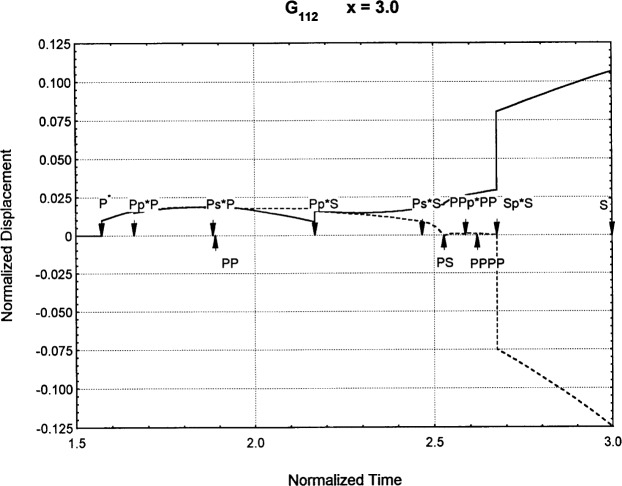
The spatial derivative of the Green’s function ***G***_112_. *X* = 3. (A) The dashed curve is the result for a liquid coupled interface. (B) The solid curve is the result for a welded interface.

**Fig. 21:**
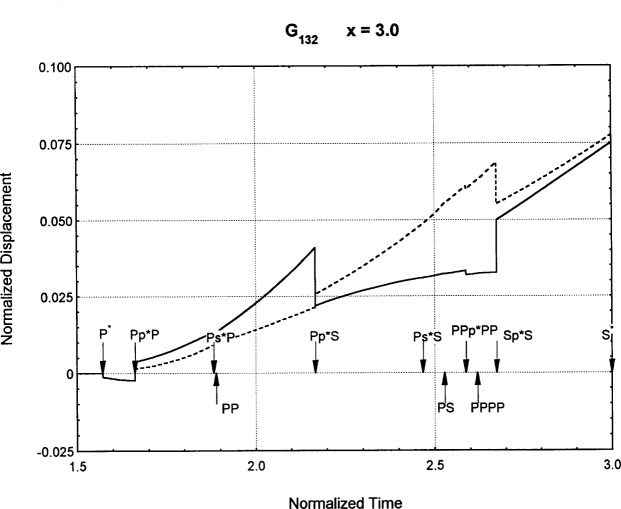
The spatial derivative of the Green’s function ***G***_132_. *X* = 3. (A) The dashed curve is the result for a liquid coupled interface. (B) The solid curve is the result for a welded interface.

**Fig. 22:**
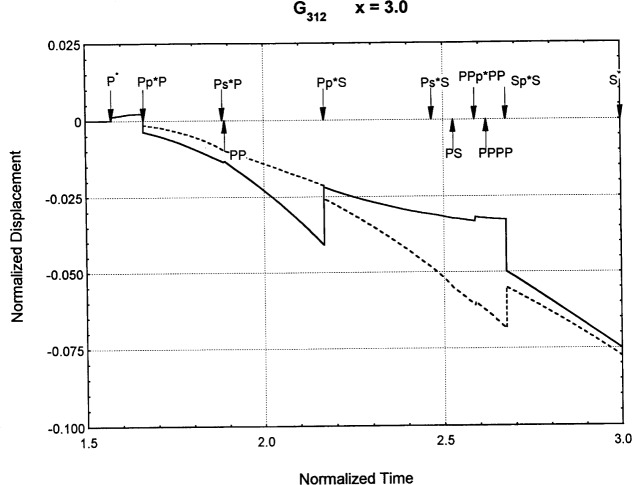
The spatial derivative of the Green’s function ***G***_312_. *X* = 3. (A) The dashed curve is the result for a liquid coupled interface. (B) The solid curve is the result for a welded interface.

**Fig. 23:**
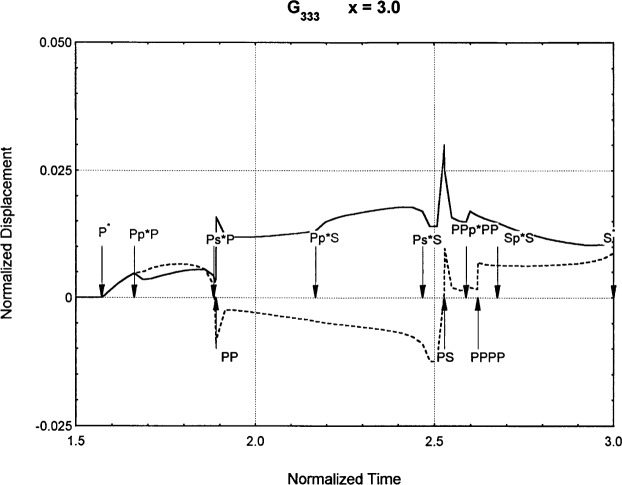
The spatial derivative of the Green’s function ***G***_333_. *X* = 3. (A) The dashed curve is the result for a liquid coupled interface. (B) The solid curve is the result for a welded interface.

**Fig. 24:**
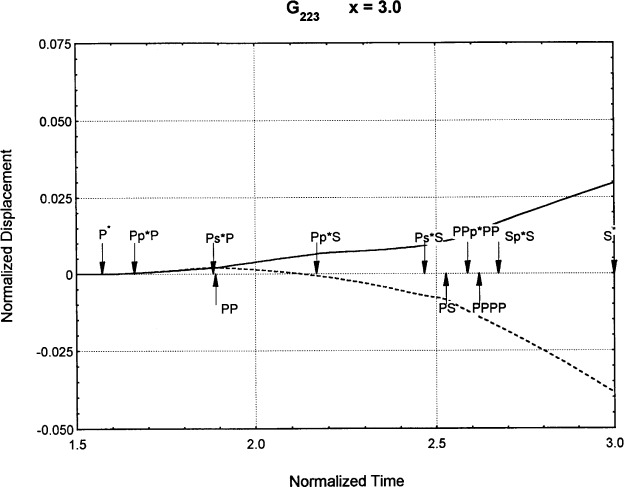
The spatial derivative of the Green’s function ***G***_223_. *X* = 3. (A) The dashed curve is the result for a liquid coupled interface. (B) The solid curve is the result for a welded interface.

**Fig. 25:**
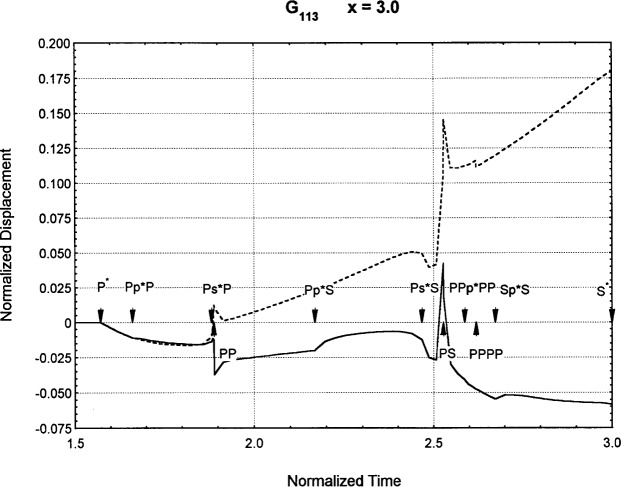
The spatial derivative of the Green’s function ***G***_113_. *X* = 3. (A) The dashed curve is the result for a liquid coupled interface. (B) The solid curve is the result for a welded interface.

**Fig. 26:**
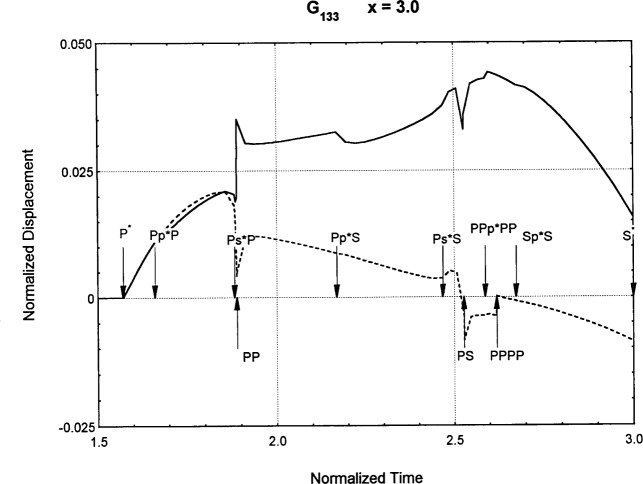
The spatial derivative of the Green’s function ***G***_133_. *X* = 3. (A) The dashed curve is the result for a liquid coupled interface. (B) The solid curve is the result for a welded interface.

**Fig. 27:**
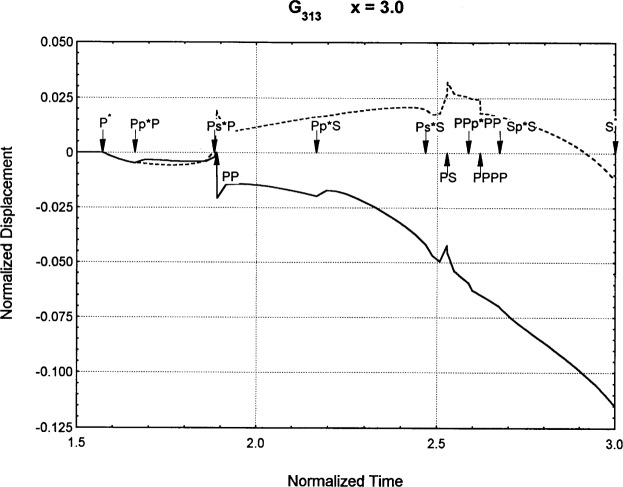
The spatial derivative of the Green’s function ***G***_313_. *X* = 3. (A) The dashed curve is the result for a liquid coupled interface. (B) The solid curve is the result for a welded interface.

Figures 6–27 show the computed results of the components of the Green’s tensor and their spatial derivatives for a plexiglass layer on a glass substrate. They were carried out on an IBM compatible personal computer. The abscissa coordinate, time, is normalized by the time required for a shear wave to vertically travel the thickness of the layer. The solid curves are the results for a welded interface condition, while the dashed curves are for a liquid coupled interface condition. The subscripts *ij* (11, 12, 13, ..etc.) or *ijk* (111…etc.) indicate the response in a specified coordinate direction, *i*, of a point detector located on the top surface of the layer to a point force with step function time dependence exerted on the top surface in a specified coordinate direction, *j*. The index *k* denotes the specific spatial derivative direction. Physically, the spatial derivative function *Gij*, *k* can be considered as the displacement in the *i*-direction due to a differentiated force which is equivalent to a couple or a dipole. The distance between the source and detector is three times the thickness of the layer (except for Figs. 7 and 8) and is chosen in such a way that the very large Rayleigh wave arrivals are avoided within the given observation time, and therefore the details of the early time arrivals can be examined. The other reason for choosing this distance is to provide a basis of comparison for our experiments which we have recently conducted [15] with a geometry that is convenient to arrange and carry out. It is seen from these figures that the differences in the results with different interface conditions are very significant. The responses for a delta function time dependence can be obtained by numerical differentiation of the responses mentioned above. The results for different distances are shown in Figs. 6–8. If we compare the early time arrivals in Fig. 6 (*x* = 3) and Fig. 7 (*x* = 6), they are very different only because of the change of the distance between the source and detector. This occurs because when the distance increases, more head waves arrive, and different arrivals change their order of occurrence. The Rayleigh wave arrivals with welded and liquid coupled interface conditions can be clearly seen from Fig. 8 and they are coincident. The early time arrivals and their differences between the two different interface conditions are totally masked by the amplitude of the Rayleigh wave arrivals. Little information about the interface can be derived.

Determination of the Green’s function for a structural configuration results in a method for determining the response of the structure to a temporally varying and spatially distributed input loading. The motion of a point on the structure can be computed due to, for example, a transient contact input force. The transient waveform input is the force that would be produced by a point source transducer. For an input waveform of interest, the motional response is calculated by a point-by-point convolution of the time differential of the source waveform with the Green’s function component that represents the step function response in the motional direction of interest.

The response of a structure due to a damped sinusoid input can be easily determined and may be of interest in analyzing the response to pulses used for probing materials and structures. The response of a layered structure interrogated by a pulsed laser beam can also be determined by convolving an input waveform with the derivative of the components of the Green’s tensor that represents a dipole source. By comparing the response in the case of a vacuum lower half-space with the results of a welded solid half-space, the behavior of a large debond can be calculated. The large differences in amplitude and polarity of the first few head waves and regular reflected rays are strong indicators that there is no bond. These results and others will be described in a future paper. Many other responses of interest for particular applications, such as nondestructive evaluation, are envisioned.

## 13. Appendix A. The Willis Inversion Method

This appendix directly quotes from Ref. [3]. Consider the inversion of transforms of the form

$$\begin{array}{l} f\left(t,x\right)=\frac{1}{8\pi^{3}}\int_{-\infty -0i}^{\infty -0i}d\omega \iint d\xi_{1}d\xi_{2}\sum F\left(\omega ,\xi \right)\\ \exp\left[-i\left(\xi_{\alpha }x_{\alpha }-\omega t+\lambda_{1}\xi_{3}^{M}+\lambda_{2}\xi_{3}^{N}\right)\right] \end{array}$$ (A.1)

where *F*(*ω*,*ξ*) is a homogeneous function of degree −2. Each term of the integrand, which represents a generalized ray in the text, is analytic for *Im*(*ω*) < 0 and the integration of each term vanishes for *t* < 0. So *f*(*t*, *x*), i.e., the integration of the sum of the integrands should vanish for *t* < 0. The functions *ξ^M^*_3_ and *ξ^N^*_3_ are homogeneous of degree 1 in *ω*, *ξ*_1_, and *ξ*_2_; λ_1_ξ*^M^*_3_ and λ_1_ξ*^N^*_3_ have imaginary parts.

Setting

$$\Omega =\omega /|\xi |,\;\eta_{\alpha }=\xi_{\alpha }/|\xi |,$$ (A.2)

where $|\xi |={\left(\xi_{1}^{2}+\xi_{2}^{2}\right)}^{1/2}$, gives

$$\begin{array}{l} f\left(t,x\right)=\frac{1}{8\pi^{2}}\underset{\varepsilon \to 0}{\lim}\int_{-\infty -0i}^{\infty -0i}d\Omega \oint_{\eta |=1}ds\sum F\left(\Omega ,\eta \right)\int_{0}^{\infty }d|\xi |\\ \exp\left[-i|\xi |\left(-\Omega t+\eta_{\alpha }x_{\alpha }+\lambda_{1}\xi_{3}^{M}+\lambda_{2}\xi_{3}^{N}-i\varepsilon \right)\right] \end{array}$$ (A.3)

where $\xi_{3}^{M}=\xi_{3}^{M}\left(\Omega ,\eta \right)$. The “convergence factor” e^−|^*^ξ^*^|^ is inserted to facilitate evaluation of the integrals in Eq. (A.3) by simple quadrature; generalized functions are continuous with respect to limiting operations of the type introduced, and the limit *ε* → 0 can be taken at any convenient stage. Evaluating the integral with respect to |*ξ*| gives

$$\begin{array}{l} f\left(t,x\right)=\frac{1}{8\pi^{2}i}\underset{\varepsilon \to 0}{\lim}\sum \oint_{\left|\eta \right|}ds\int_{-\infty -0i}^{\infty -0i}\;\\ d\Omega \left(\frac{F\left(\Omega ,\eta \right)}{-\Omega \tau +\eta_{\alpha }x_{\alpha }+\lambda_{1}\xi_{3}^{M}+\lambda_{2}\xi_{3}^{N}-i0}\right). \end{array}$$ (A.4)

Setting

$$\phi =-\Omega t+\eta_{\alpha }x_{\alpha }+\lambda_{1}\xi_{3}^{M}+\lambda_{2}\xi_{3}^{N}-i\varepsilon$$ (A.5)

the integral with respect to *Ω* is now evaluated by closing the contour in the lower half-plane because the integrand has behavior as *O*(*Ω*^−2^) for large *Ω* and using Cauchy’s theorem. This gives

$$f\left(t,x\right)=\frac{-1}{4\pi^{2}}\sum \oint_{\begin{array}{l} |\eta |=1\\ \phi =0 \end{array}}ds{\frac{F\left(\Omega ,\eta \right)}{-t+\lambda_{1}\xi_{3,\Omega }^{M}+\lambda_{2}\xi_{3,\Omega }^{N}}|}_{\phi =0},$$ (A.6)

where *Ω* is now taken as the root in the lower half-plane of the equation

$$-\Omega t+\eta_{\alpha }x_{\alpha }+\lambda_{1}\xi_{3}^{M}\left(\Omega ,\eta \right)+\lambda_{2}\xi_{3}^{N}\left(\Omega ,\eta \right)=0i,$$ (A.7)

if there is such a root. If there is more than one root, then the contribution from each root should be included; if none, then that term of the integrand is replaced by zero.
